# Microbial community structure and niche differentiation under different health statuses of *Pinus bungeana* in the Xiong'an New Area in China

**DOI:** 10.3389/fmicb.2022.913349

**Published:** 2022-09-02

**Authors:** Jia Yang, Abolfazl Masoudi, Hao Li, Yifan Gu, Can Wang, Min Wang, Zhijun Yu, Jingze Liu

**Affiliations:** ^1^Hebei Key Laboratory of Animal Physiology, Biochemistry and Molecular Biology, Hebei Collaborative Innovation Center for Eco-Environment, Ministry of Education Key Laboratory of Molecular and Cellular Biology, College of Life Sciences, Hebei Normal University, Shijiazhuang, China; ^2^School of Geographic Sciences, Hebei Normal University, Shijiazhuang, China

**Keywords:** *Pinus bungeana*, microbiota, rhizosphere, niche differentiation, plant–microbe interactions

## Abstract

*Pinus bungeana* is a native but endangered plant species in China, with high ornamental value and adaptability to drought and cold. The relationship between the soil community structure and endophytic microbes in the tissues of *P. bungeana* under different health statuses is poorly understood. In this study, the endophytic bacterial and fungal communities of *P. bungeana* under different health statuses were compared and analyzed in the Xiong'an New Area. Using high-throughput deep sequencing [16S and internal transcribed spacer (ITS) rRNA] techniques, the effect of the health status of *P. bungeana* on the microbial communities in bulk soil, rhizospheric soil, roots, stems, and leaves was determined in this study. We observed that the diversity of the bacterial and fungal communities of the aboveground parts (stems and leaves) of healthy *P. bungeana* plants was much higher than that of the unhealthy plants. However, the diversity of bacterial and fungal communities in the belowground parts (bulk soil, rhizospheric soil, and roots) showed almost no difference in microbial community richness, indicating that the possible cause of illness was transmitted in a “top-down” manner. Furthermore, there were significant differences in the microbial diversity and community structure in different ecological niches of *P. bungeana* (*P* < 0.01). Proteobacteria and Actinobacteria were the dominant bacterial phyla, while Ascomycota, Basidiomycota, and Mortierellomycota were the predominant fungal phyla. Redundancy analysis (RDA) revealed that soil organic matter (SOM), total phosphorous (TP), total potassium (TK), total nitrogen (TN), water content (WC), power of hydrogen (pH), total carbon (TC), and the ratio of carbon to nitrogen (C/N) were significantly correlated with the composition of the microbial communities. Altogether, these results provide a scientific basis for further studies on the mechanism underlying the “aboveground–underground” microbial interactions in plantation forests, which can aid in promoting the healthy and sustainable development of the Millennium Xiulin forest in the Xiong'an New Area.

## Introduction

Plants and their endophytic microflora usually form a “holobiont” with complicated interactions and evolution (Vandenkoornhuyse et al., 2015; Hassani et al., 2018). Interactions between host plants and microbiota can be divided into three categories, namely, negative (pathogenic), positive, and neutral interactions (Zheng and Gong, 2019). Of these interactions, symbiosis is considered synergistic and aids both interacting partners (Lopez et al., 2012). For instance, it has been reported that microbial communities play a crucial role in promoting the growth and development of *Arabidopsis thaliana* (Durán et al., 2018), grapevine (Rolli et al., 2015), and citrus root (Zhang et al., 2017); improving plant resistance; and promoting sustainable and healthy production (Weyens et al., 2009; Brader et al., 2014; Naveed et al., 2014). Furthermore, microbial populations can improve nutrient transport and utilization by host plants and promote host resistance against biotic and abiotic stresses. The host plants, in turn, provide a stable niche habitat, energy, and carbon source for the microbial population (Raaijmakers et al., 2009; Peiffer et al., 2013; Philippot et al., 2013).

Each plant compartment, including roots, stems, leaves, flowers, and seeds, represents a unique niche of microbial entities and possesses unique microbial communities (Lindow and Brandl, 2003). Moreover, the endophytic microbiota shows significant variations in different tissues and organs (Ottesen et al., 2013). Soil is rich in microflora (Zarraonaindia et al., 2015; Mangeot-Peter et al., 2020); for the successful colonization of plant tissues, soil microbiota needs to overcome the immune system of the host plant and abiotic stresses, following which some populations are eliminated from the internal microenvironment of the plant due to the lack of adaptation (Bulgarelli et al., 2013). In addition, the plant microbial community is required to adapt to the varying climatic and soil conditions. The changes in the soil microenvironment depend on specific microbiota, soil properties (pH and nutrients), soil type, light, temperature, humidity, and other environmental factors. This alteration can significantly affect plant roots and endophytic flora (Delgado-Baquerizo et al., 2018; Semenov et al., 2018; O'brien et al., 2019; Wang et al., 2021). In most cases, the microorganisms in underground tissues differ from those in the aboveground parts of plants. It has been reported that the microbiota in the aboveground parts of mature poplar is markedly different from that in the rhizospheric soil (Gottel et al., 2011). Another study demonstrated that the microbial species composition and diversity of the underground parts of *Cannabis sativa* are significantly different from those in the aboveground tissues (Wei et al., 2021).

In plant–soil–microorganism systems, the interaction between microbiota can affect or directly change the health status of host plants. For instance, the resistance of *A. thaliana* and apple fruits against *Botrytis cinerea* was found to be highly associated with the plant symbiont, *Pseudomonas* sp. (Unyarat et al., 2016). Another study reported that the application of *Actinomyces* sp. to nursery plants effectively reduces infection with pathogenic bacteria in the roots (Álvarez-Pérez et al., 2017). It has been reported that soil biota might protect against infections with fungal pathogens inoculated in plant roots (Rodrigo et al., 2011).

*Pinus bungeana* Zucc. Ex Endl. (*P. bungeana*) is an endangered tree species and the only three-leaf pine in East Asia. With its beautiful posture and unique bark, the timber of *P. bungeana* can be used to construct houses, furniture, stationery, and other materials, which have great ornamental and economic value. *P. bungeana* is an important species for ecological construction, with robust adaptability to dry, cold, calcareous, and mild saline–alkali soils. The plant has exceptional resistance to sulfur dioxide, hydrogen fluoride, and smoke dust pollution and plays a crucial role in forest regeneration, thereby serving as a unique tree species for the creation of environmental protection forests in industrial and mining areas (Syring et al., 2007; Yang et al., 2015; Guo et al., 2020). In a previous study, we observed highly diversified microflora in the roots of *P. tabuliformis* (Wang et al., 2020). However, the microbial community structure and niche differentiation in different tissues of *P. bungeana* in the man-made Xiong'an New Area forest remain to be elucidated.

Accumulating studies have demonstrated that plants can significantly influence the structure of the plant microbiome (Esmaeili et al., 2017; Yu and Hochholdinger, 2018) by producing different types of root exudates (Jacoby et al., 2017). However, there is a paucity of studies on the impact of the varying plant health status on the microbial communities in plantation forests. We, therefore, hypothesized that endophytic microbial communities are unique in different compartments of *P. bungeana*, and the health status of the plants shapes the microbial community structure and niche differentiation in different tissues. Therefore, in this study, the bacterial and fungal communities in the rhizospheric soil, bulk soil, and the inner layers of roots, stems, and leaves of *P. bungeana* plants of different health statuses were explored using 16S rRNA and internal transcribed spacer (ITS) gene sequencing techniques, for elucidating the complex interactions between microorganisms and the host plant. These findings will provide a scientific basis for further studies on the mechanism of “aboveground–underground” microbial interactions in plantation forests, which can aid in promoting the healthy and sustainable development of the Millennium Xiulin forest in the Xiong'an New Area.

## Materials and methods

### Sampling site and sample collection

The sampling sites were located in the Xiong'an New Area (38° 43′ to 39° 10′ N and 115° 38′ to 116° 20′ E) announced by the government of the People's Republic of China on April 1, 2017. As part of the development of the new national region, the Xiong'an New Area is another key area after the Shenzhen Special Economic Zone and the Shanghai Pudong New Area (Li et al., 2020). The Xiong'an New Area has a warm temperate continental monsoon-type climate with four seasons and significant differences between cold, warm, and humid periods and has an annual average temperature of 12.1°C, frost-free period of 173 days, and an average yearly precipitation of 560 mm (Xu et al., 2018; Xie et al., 2019). The Millennium Xiulin area was launched in November 2017 with the aim of creating an appealing ecological environment and constructing a new and impressive ecological city. *P. bungeana* is one of the main afforestation tree species in the Millennium Xiulin area.

In total, three blocks were selected in the Millennium Xiulin area, and healthy *P. bungeana* plants (H, with dark green leaves and no sign of pests or diseases) and unhealthy *P. bungeana* plants (U, with withered needles and yellow caducous, leaves with tan spots) were randomly selected from each block. In addition, the unhealthy plants in the study area were characterized by the mixed occurrence of brown spots, needle cast, and needle blight, which could have been caused by pathogens, a mild climate, high humidity, or poor management of the barren soil. A total of 30 samples, including healthy bulk soil (HFRS), healthy rhizospheric soil (HRS), healthy roots (HR), healthy stems (HS), healthy leaves (HL), unhealthy bulk soil (UFRS), unhealthy rhizospheric soil (URS), unhealthy roots (UR), unhealthy stems (US), and unhealthy leaves (UL), were collected from the experimental area in the September of 2021, and each sample was collected in triplicate. After removing the surface impurity, the bulk and rhizospheric parts of coniferous *P. bungeana* plants were collected. Underground root samples with a diameter of ~0.3 cm were extracted from depths of 0–30 cm, and stems with diameters of ~0.5 cm were selected as stem samples. The rhizospheric soil was strictly defined as the soil particles that adhered to the roots. The bulk soil was collected from the topsoil region (0–15 cm) and from regions more than 20 cm away from the roots. The leaf chamber represented all the leaves at the end and sub-end of the stem branch, which were collected using sterile scissors. Disposable gloves were worn and replaced every time different tissues were sampled. The samples were placed in a portable refrigerator, transported to the laboratory at low temperature (~−20°C), and stored at −80°C for further analyses.

### Sample processing

The samples were processed in our laboratory according to the method described by Beckers et al. (2016). In brief, the bulk soil samples were processed by removing the plant debris and stones by sieving (<2 mm pore size) and divided into two groups: one group was air-dried and used for determining the physicochemical properties, while the other group was placed in a sterile bag and stored at −80°C. The whole root system was depleted of soil particles by shaking on a platform for 20 min at 120 rpm, and the soil particles that were directly removed from the roots represented the rhizospheric samples. The root, stem, and leaf compartments were first rinsed with sterile ddH_2_O for 30 s and subsequently rinsed with 70% (v/v) ethanol and sodium hypochlorite (2.5% sodium hypochlorite with 0.1% Tween 80) for 2 and 5 min, respectively. The tissues were then washed with 70% (v/v) ethanol for an additional 30 s to remove the epiphytic bacteria and fungi from the surface, following which the samples were rinsed five times with sterile ddH_2_O (Beckers et al., 2017). The plant samples were cut into small pieces with a sterile scalpel and then sterilized with sterile PBS (130 mM NaCl, 7 mM Na_2_HPO_4_, 3 mM NaH_2_PO_4_, at pH 7.4). After sterilization, the plant samples were homogenized and subjected to laminar airflow under sterile conditions. Finally, quadruple aliquots (1.5 mL) of homogeneous plant samples (roots, stems, and leaves) from each sample were stored at −80°C until DNA extraction (Wagner et al., 2016).

### DNA extraction

The total DNA was extracted from the bulk soil (0.4 g) and rhizospheric soil (0.4 g) of *P. bungeana* using a FastDNA^Ⓡ^ Spin Kit (MP Biomedicals, USA), according to the standard operating instructions. All the plant tissues, including roots, stems, and leaves, were cut into fine pieces (<5 mm), and 50 mg of the tissues were ground for 3 min in liquid nitrogen using 5-mm beads. The DNA was extracted from the pulverized tissue using the same kit as previously described. The extraction was repeated two times to obtain sufficient DNA yield. The concentration and purity of the DNA were measured using a NanoDrop 2000 spectrophotometer (Thermo Fisher Scientific, Wilmington, DE, USA), and the integrity of the DNA was subsequently examined by agarose gel electrophoresis with 1% agarose gels (Cregger et al., 2018; Wang et al., 2019).

### Polymerase chain reaction amplification and Illumina MiSeq sequencing

PCR amplification was performed in our laboratory, and Illumina MiSeq sequencing was performed by Majorbio company (Majorbio Bio-Pharm Technology Co., Ltd., Shanghai, China). The bacterial 16S rRNA amplicon library was generated by two-step PCR, and the first round of PCR amplification was performed using 799F (5'-AACMGGATTAGATACCCKG-3') and 1392R (5'-ACGGGCGGTGTGTRC-3') primers (Bodenhausen et al., 2013; Hartman et al., 2018). PCR amplification of the 16S rRNA gene was performed under the following conditions: initial denaturation at 95°C for 3 min, followed by 27 cycles of denaturation at 95°C for 30 s, annealing at 55°C for 30 s, extension at 72°C for 45 s, and final extension at 72°C for 10 min with a hold step at 4°C. The PCR mixtures contained 5 × 4 μL of TransStart FastPfu buffer, 2 μL of 2.5 mM dNTPs, 0.8 μL forward primer (5 μM), 0.8 μL reverse primer (5 μM), 0.4 μL TransStart FastPfu DNA polymerase, 10 ng template DNA, and up to 20 μL ddH_2_O. The PCR was performed in triplicate. The PCR product was separated on 2% agarose gels and purified using an AxyPrep DNA Gel Extraction Kit (Axygen Biosciences, Union City, CA, USA), according to the manufacturer's instructions. The purified amplicons were next subjected to the second round of PCR amplification using 799F (5'-AACMGGATTAGATACCCKG-3') and 1193R (5'-ACGTCATCCCCACCTTCC-3') primers to reduce the length of the amplicon (394 bp) for sequencing. The PCR conditions were the same as those used in the first round, with the exception that the number of cycles was reduced to 13. The products were purified using an AxyPrep DNA Gel Extraction Kit (Axygen Biosciences, Union City, CA, USA) and quantified using a Quantus™ Fluorometer (Promega, Madison, WI, USA).

The universal primers, ITS1F (5'-CTTGGTCATTTAGAGGAAGTAA-3') and ITS2R (5'-GCTGCGTTCTTCATCGATGC-3'), were selected for generating the fungal ITS amplicon library. The reaction system, purification, and quantification procedures were the same as those used for bacterial PCR. Purified amplicons were pooled in equimolar concentrations and subjected to paired-end sequencing using the Illumina MiSeq PE300 platform (Illumina, San Diego, CA, USA) by Majorbio company (Majorbio Bio-Pharm Technology Co., Ltd., Shanghai, China). Bacterial and fungal raw data were deposited at the National Center for Biotechnology Information Sequence Read Archive database under the BioProject numbers PRJNA821431 (accession number: SUB11251518) and PRJNA821418 (accession numbers: SUB11250928), respectively.

### Bioinformatics analyses

Bioinformatics analyses were performed by Majorbio Bio-Pharm Technology Co., Ltd. (Shanghai, China). The raw reads were demultiplexed, quality-filtered, and controlled using fastp version 0.20.0 (Chen et al., 2018) and merged using FLASH software, version 1.2.7 (Magoč and Salzberg, 2011), with the following procedure: (i) 300-bp reads were truncated at any site with an average quality score of <20 over a 50-bp sliding window. Truncated reads shorter than 50 bp and reads containing ambiguous characters were discarded. (ii) Overlapping sequences longer than 10 bp were assembled according to their overlapped sequence. The maximum mismatch ratio of the overlapping region was set to 0.2. The reads that could not be assembled were discarded. (iii) The samples were distinguished according to the barcode and primer sequences, and the sequence direction was adjusted for exact barcode matching and two nucleotide mismatches in primer matching.

The operational taxonomic units (OTUs) were clustered using UPARSE software, version 7.1. In order to eliminate the redundant computational steps in the intermediate processes of analysis, the non-repetitive sequences were extracted from the optimized sequences, and single non-repetitive sequences were removed. The non-repetitive sequences (excluding single sequences) were clustered based on the OTUs using a similarity threshold of 97%, and chimeras were removed during clustering to obtain the representative OTU sequences (Dickie, 2010). Chimeric sequences were identified and removed using UCHIME software, version 3 (Edgar et al., 2011), against the SILVA database (version 138), the 16S rRNA database for bacteria, and the UNITE ITS database (version 7.0) for fungi, using a confidence threshold of 70% (Wang et al., 2019). Non-bacterial and non-fungal sequences were removed from the data set (Wang et al., 2022).

### Statistical analyses

All the statistical analyses were performed using a cloud platform (Majorbio Bio-Pharm Technology Co., Ltd.). Analysis of variance (ANOVA), Student's *t*-test, and *post-hoc* analyses were conducted using R version 3.5.1 (R Core Team, 2018). Statistical analyses of the rhizospheric and bulk soil data of healthy and unhealthy *P. bungeana* plants, and correlation analysis of soil and microbial communities were performed using SPSS version 22.0 for Windows (IBM Corp, NY, Armonk, USA). The *post-hoc* comparison was performed to measure significant differences in microbial richness and composition among different compartments in healthy and unhealthy plants by Tukey's honestly significant difference (HSD) test. QIIME version 1.9.1 was used to calculate the beta diversity distance, and R version 3.5.1 was used for Bray–Curtis hierarchical clustering and PCoA statistical analysis to identify the similarity or difference of the community structure among different samples. The similarities and differences in the relationships of microbial community structures in different compartments of *P. bungeana* were investigated with heatmaps generated using the VEGAN package. Linear discriminant analysis (LDA) coupled with effect size measurements (LEfSe) analysis was conducted to identify the statistically different biomarkers between groups (Wei et al., 2021). The alpha diversity indices (Chao, Shannon, Simpsoneven) were calculated using mothur version 1.30.2 (Schloss et al., 2011). Calculation of beta diversity and generation of Venn diagrams were performed using R version 3.5.1. The Circos table viewer was used for visualizing the Circos diagram, which depicted the distribution proportion of core microbiota in each of the plant compartments and provided a measure of the distribution proportion of each dominant species in the different samples (Krzywinski et al., 2009). Network visualization was performed to obtain information regarding the species abundance in the different compartments using Cytoscape version 3.4.0 (Shannon et al., 2003). The co-existing relationships among the species in the different niches were simultaneously determined for elucidating the similarities and differences between samples.

### Analyses of the physicochemical properties of soil

The pH of the soil samples was measured from a soil–water suspension (1:5 *m*/*v*) using a Benchtop FiveEasy Plus™ pH Meter (Mettler Toledo, Shanghai, China). The total carbon (TC), total nitrogen (TN), and the ratio of carbon to nitrogen (C/N) were determined using an elemental analyzer (Vario EL-3, Elementar, Germany). The soil organic matter (SOM) was measured using the dichromate oxidation method (Certini et al., 2007). The water content (WC) was determined using the drying method, while total soil phosphorus (TP) and total potassium (TK) were measured using the acid solution molybdenum-antimony colorimetric method and a flame photometer, respectively, according to China's Soil Standard protocols for forest regions (LY/T 1232–2015, LY/T 1234–2015, and LY/T 1228–2015, in Chinese).

## Results

### Alpha diversity of microbial taxa in different plant niches and soil

A total of 593,512 high-quality reads were obtained for the bacterial species, with an average length of 377 bp, while 2,247,150 reads were obtained for the fungal species, with an average length of 236 bp. A total of 1,777 bacterial and 1,978 fungal OTUs were identified across all the samples. The rarefaction curves for evaluating the richness of the bacterial OTUs were generally close to saturation, indicating that the volume of the sequenced reads was reasonable (Supplementary Figure S1). The rarefaction curves showed that the alpha diversity of the fungal lineages for the HL and HS samples was higher than that of the other eight samples.

**Figure 1:**
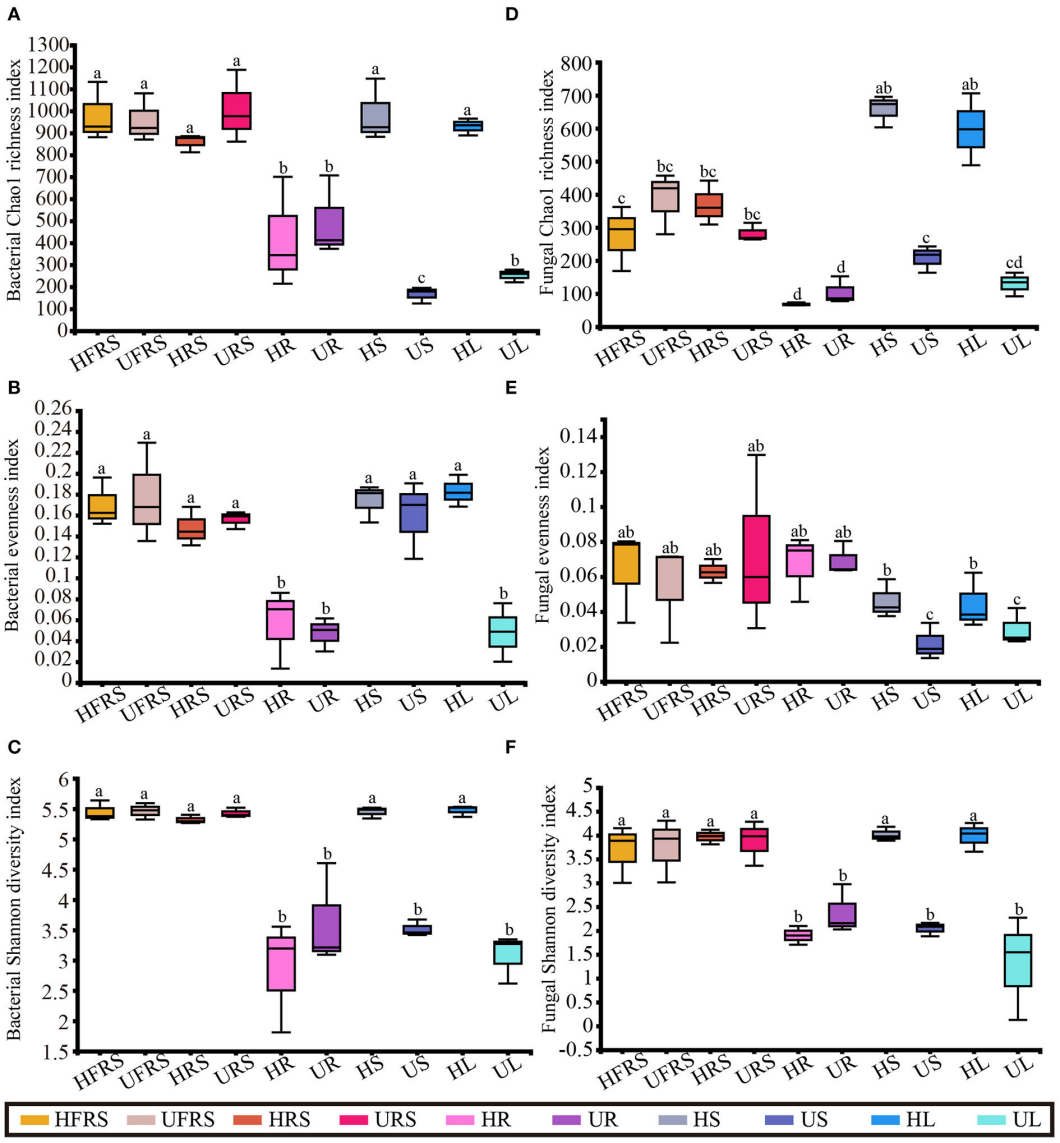
Microbial alpha diversity in healthy and unhealthy *P. bungeana* plants. **(A)** Bacterial Chao1 richness index. **(B)** Bacterial evenness index. **(C)** Bacterial Shannon diversity index. **(D)** Fungal Chao1 richness index. **(E)** Fungal evenness index. **(F)** Fungal Shannon diversity index. All the diversity indices were calculated at the OTU level. The box plots depict the first (25%) and third (75%) quartiles, the median, mean, and the maximum and minimum observed values within each data set. The alpha diversity estimates represent the results of three biological replicates for each group. The data were analyzed by one-way ANOVA and Tukey–Kramer *post-hoc* comparisons. Significant differences (*P* < 0.05) across plant compartments are indicated with lowercase letters. HFRS, healthy bulk soil; HRS, healthy rhizosphere soil; HR, healthy roots; HS, healthy stems; HL, healthy leaves; UFRS, unhealthy bulk soil; URS, unhealthy rhizosphere soil; UR, unhealthy roots; US, unhealthy stems; UL, unhealthy leaves.

There was no noticeable difference in the bacterial richness among the HFRS, UFRS, HRS, URS, HS, and HL samples, and the bacterial richness of these samples was significantly higher than that of the other samples (*P* < 0.01). There was no significant difference in bacterial richness between the HR and UR samples and between the US and UL samples, which showed the lowest bacterial richness (Figure 1A). There was no significant difference in the bacterial evenness among the samples in the same niche, with the exception of the UL sample, in which the bacterial evenness was significantly lower than that of the HL samples. This indicated that bacterial evenness was affected by differences in health conditions (*P* < 0.01). Bacterial evenness was lowest in the root and UL samples (Figure 1B). The bacterial diversity in the rhizospheric soil, bulk soil, and stems and leaves of healthy *P. bungeana* plants was significantly higher than that of the other samples (*P* < 0.01). The bacterial diversity was lowest in the US, UL, and all root samples (Figure 1C).

There were no significant differences in fungal richness between the bulk and rhizospheric soil samples of *P. bungeana* at different health conditions (*P* > 0.05). In addition, there were no significant differences in the endophytic fungal communities in the roots of *P. bungeana* at two different health conditions, in different tissue niches (*P* > 0.05). The richness of the OTUs of the HS and HL samples was significantly higher than that of other samples (*P* < 0.01), whereas that of the HFRS, UFRS, HRS, and URS samples was substantially higher than that of the root samples (*P* < 0.01). There was no noticeable difference in the richness of the OTUs among all the root samples and among the UL, US, and UL samples (Figure 1D). There was no apparent difference in the fungal evenness in the same niches between the healthy and unhealthy plants, while the evenness in the US and UL samples was significantly lower than that of the HFRS, UFRS, HRS, URS, HR, and UR samples (*P* < 0.01) (Figure 1E). The fungal diversity of the HFRS, UFRS, HRS, URS, HS, and HL samples was significantly higher than that of the other samples (*P* < 0.01), and there were no significant differences in the fungal diversity among all the root samples and the US and UL samples (Figure 1F).

### Beta diversity of microbial taxa in different plant niches and soil

**Figure 2:**
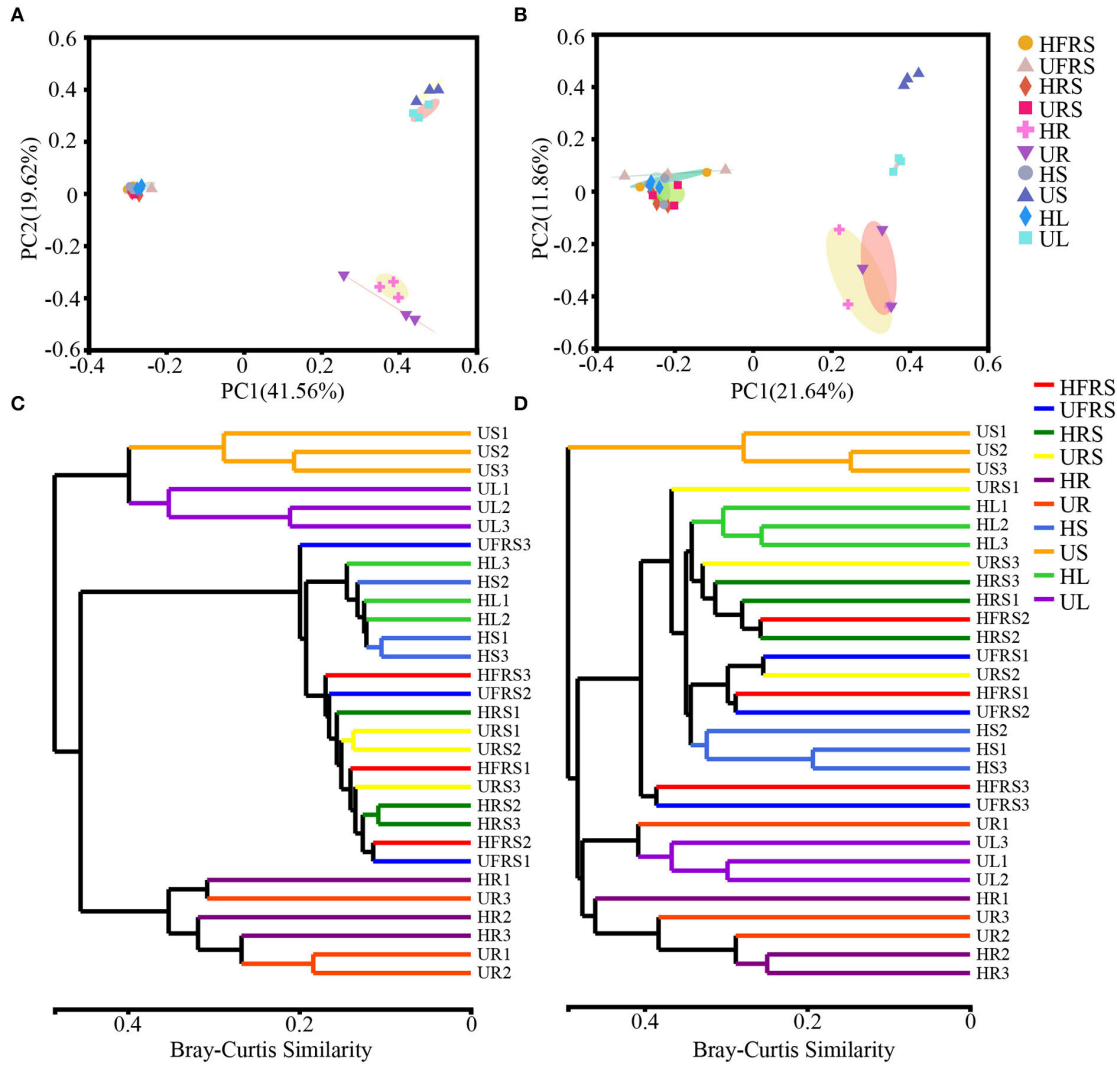
Composition of the microbial communities in the rhizospheric and bulk soil samples and in different plant compartments (roots, stems, and leaves) of healthy and unhealthy *P. bungeana* plants was analyzed by principal coordinates analysis (PCoA) and dendrograms constructed using the hierarchical clustering algorithm at the OTU level. PCoA of bacterial **(A)** and fungal **(B)** community compositions plotted based on Bray–Curtis distance. The percent of variation explained by PC1 and 2 is given as percent values at respective axes. **(C)** Bacterial and **(D)** fungal dendrograms constructed by hierarchical clustering of microbial communities. PCoA was based on the rarefaction curve of the minimum sample sequence number. Hierarchical clustering was based on the Bray–Curtis distance and superimposed on PCoA plots. The OTUs were constructed using a sequence similarity threshold of 97%. Abbreviations of variables are given in Figure 1.

The bacterial beta diversity was evaluated at the phylum and OTU levels, while fungal beta diversity was evaluated at the class and OTU levels. Principal coordinate analysis (PCoA) revealed general similarities in the structures of bacterial and fungal communities among the samples (Figure 2). The microbial communities in the roots of *P. bungeana* were distinguishable, regardless of the health status of the plants. In addition, the US and UL microbial communities were separated from those of the other samples at the OTU and class levels, while the microbial communities in the HFRS, UFRS, HRS, URS, HS, and HL samples were indistinguishable. PC1 and PC2 explained 41.56 and 19.62%, respectively, of the total bacterial variation at the OTU level (Figure 2A). PC1 and PC2 explained 21.64 and 11.86%, respectively, of the total variation in fungi (Figure 2B). Hierarchical clustering of bacteria and fungi (at OTU, phylum, or class levels) revealed that the microbial communities from the root, stem, and leaf tissue samples were well clustered according to the respective plant compartments. By contrast, the other samples were not well clustered according to the respective compartments (Figures 2C,D; Supplementary Figure S2).

### Microbial community structure in plant niches and soils

**Figure 3:**
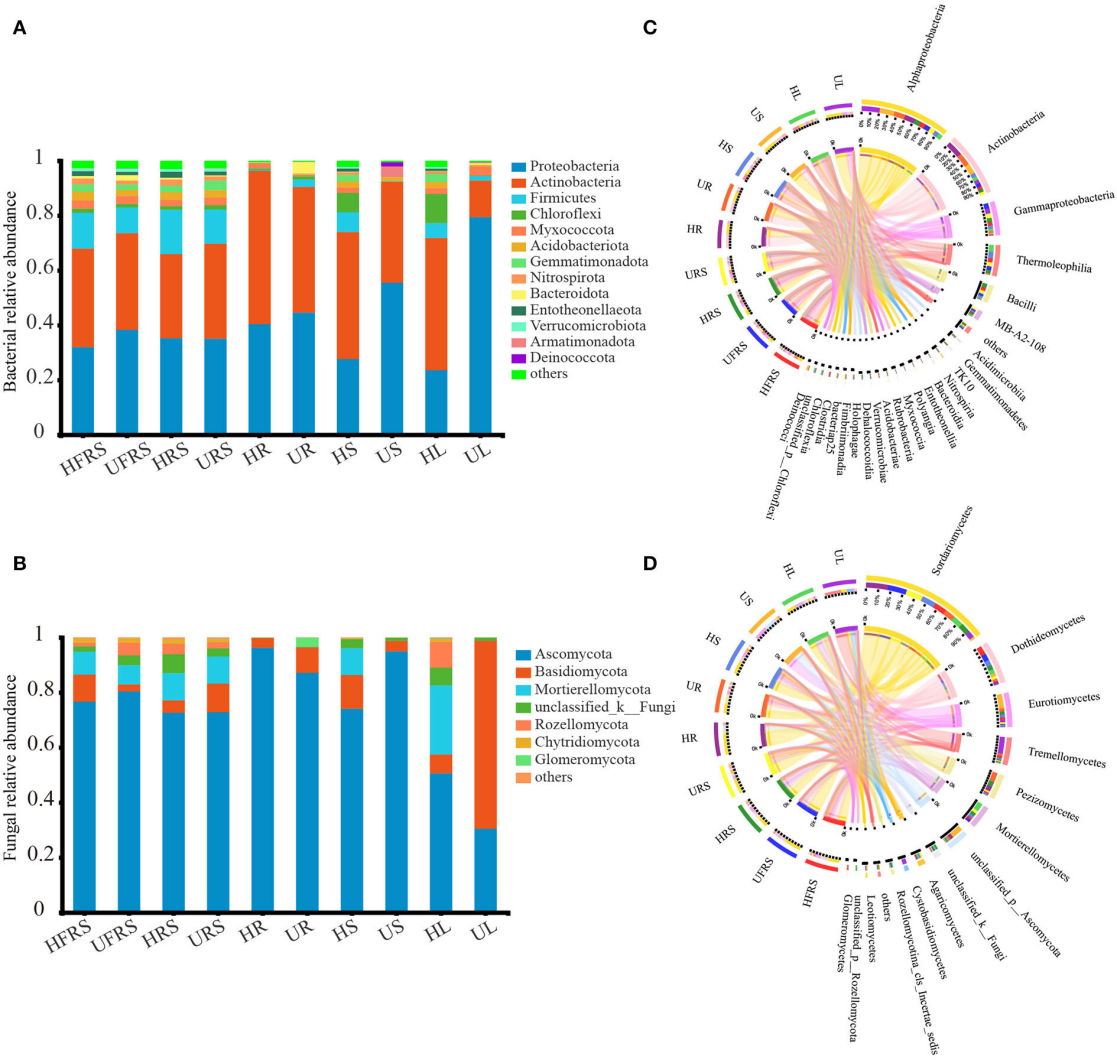
Phylum and class distributions of the OTUs. Relative sequence abundance of **(A)** bacterial and **(B)** fungal phyla associated with the soil (rhizospheric and bulk soil), and different plant compartments (roots, stems, and leaves) of healthy and unhealthy *P. bungeana* plants. The Circos plot depicts the distribution proportion of core **(C)** bacterial and **(D)** fungal classes in soil samples and different compartments of healthy and unhealthy *P. bungeana* plants. The core represents the microbes with major contributions. The Circos plot depicts the proportion of core microbiota in each compartment and reflects the proportion of each dominant species in different samples. The thickness of the ribbons in the Circos plots thickness represents the abundance of the taxon. The absolute tick above the inner segment and relative tick above the outer segment represent the read abundances and relative abundances of the taxa, respectively. Abbreviations of variables are given in Figure 1.

A total of 31 bacterial phyla were detected in the soil samples (bulk and rhizospheric soil samples) and the niches in the different compartments (roots, stems, and leaves) of *P. bungeana* under different health statuses (Figure 3A). Proteobacteria (41.16%) and Actinobacteria (38.33%) were the most abundant phyla in each of the plant niches. The major phyla in bulk and rhizospheric soils were similar, with Proteobacteria (35.12%), Actinobacteria (34.18%), and Firmicutes (12.76%) being the most abundant phyla. The frequency of endophytic bacteria in the roots, stems, and leaves of *P. bungeana* was different. The abundance of Actinobacteria was the highest in HR samples (55.95%) and lowest in UR samples (4.27%), while Bacteroidota was the most abundant phylum in all the samples. The abundance of Chloroflexi was higher in the HS (7.00%) and HL (10.26%) samples than in the other samples, while Proteobacteria was the most abundant phyla in the US (55.49%) and UL (79.41%) samples. Of the 80 bacterial classes, Alphaproteobacteria, Actinobacteria, Gammaproteobacteria, and Thermoleophilia had a higher abundance, accounting for more than 60% of the total bacteria. Alphaproteobacteria (HR = 36.57%, UR = 37.91%) and Actinobacteria (HR = 54.46%, UR = 43.31%) were the dominant bacterial classes in the roots. Thermoleophilia was the most dominant in the stems (HS = 22.79%) and leaves (HL = 25.07%) of healthy *P. bungeana* plants (Figure 3C).

There were 14 fungal phyla in the bulk soil, rhizospheric soil, root, stem, and leaf samples of *P. bungeana* (Figure 3B). Ascomycota (75.64%), Basidiomycota (8.76%), and Mortierellomycota (7.82%) were the most abundant phyla in the bulk and rhizospheric soil samples. The abundance of endophytic fungi was different among the roots, stems, and leaves of *P. bungeana*, whereas the abundance of Ascomycota was highest in the HR sample, being 96.28%. The abundance of Basidiomycota (9.30%) and Glomeromycota (3.41%) was higher in the UR samples than in the HR samples, and the abundance of Mortierellomycota was higher in the HS and HL samples than in the other tissues. The abundance of Mortierellomycota (25.12%) was highest in the HL samples. Basidiomycota was the most dominant phylum in the UL samples (68.39%). The differences among the bulk soil samples were insignificant at the class level (*P* > 0.05). Sordariomycetes, Dothideomycetes, and Eurotiomycetes were the main fungal communities (Figure 3D), representing nearly 70% of all fungal profiles in the samples. The abundance of Pezizomycetes was higher in the HRS samples (15.94%), while the abundance of Agaricomycetes was higher in the URS samples (7.15%) than in the other soil samples. The relative abundance of Sordariomycetes in the endophytic fungal communities in the HR, HS, and HL samples was low, but its abundance was higher than in the UR, US, and UL samples. Pezizomycetes accounted for 29.88% of the fungal classes in the UR samples, which was higher than that observed in the other tissues. The dominant fungal classes in the US samples were Eurotiomycetes (43.25%) and Unclassified_p_ascomycota (36.11%). Mortierellomycetes accounted for 25.08% of the fungal classes in the HL samples, which was higher than that in the other tissues. In comparison, Tremellomycetes (51.10%) and Sordariomycetes (18.60%) were the dominant classes in the UL samples. The total relative abundance of all the orders, families, and genera in relation to their distribution in the different plant compartments is depicted in Supplementary Figure S3.

### Differences in the distribution of microbial communities at the OTU level in plant niches and soil

**Figure 4:**
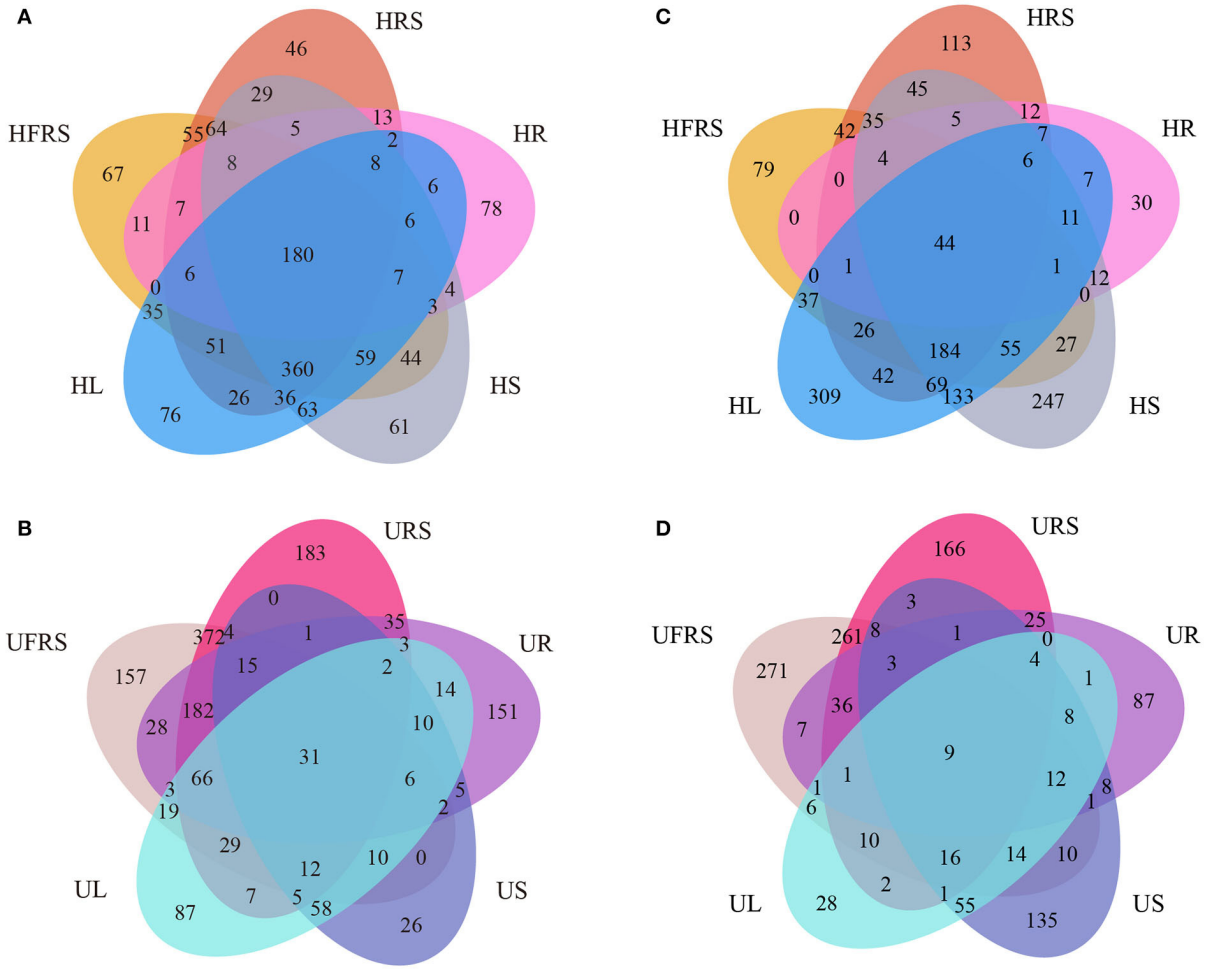
Venn diagrams depicting the unique and shared **(A,B)** bacterial and **(C,D)** fungal OTUs in different compartments of *P. bungeana*. Venn diagram depicting the bacterial OTUs in different niches of **(A)** healthy and **(B)** unhealthy *P. bungeana* plants. Venn diagram depicting the fungal OTUs in different niches of **(C)** healthy and **(D)** unhealthy *P. bungeana* plants. Abbreviations of variables are given in Figure 1.

A total of 180 bacterial OTUs (4.44%) were shared among the five samples of healthy *P. bungeana* tissues (Figure 4A). Unique OTUs were most abundant in the HFRS samples (957, 23.60%) and least abundant in the HR samples (344, 8.48%). Unique OTUs were least abundant in the HRS samples (896, 22.10%) compared to that in the HS (937, 23.11%) and HL (921, 22.71%) samples. The total number of OTUs shared among unhealthy *P. bungeana* samples was 31 (10.38%) (Figure 4B). Unique OTUs were most abundant in the URS samples (947, 31.71%) and least abundant in the US samples (187, 6.26%) compared to that of the UFRS (936, 31.35%), UR (554, 18.55%), and UL (362, 12.12%) samples. Analyses of the total OTUs in the same niche under different health conditions revealed that the aboveground tissues, including leaves (12.84%) and stems (6.64%) had less OTUs. By contrast, the underground niches, including rhizospheric soil (65.89%), bulk soil (64.61%), and roots (33.63%), had more OTUs (Supplementary Figures S4A–E).

A total of 44 fungal OTUs (1.41%) were shared among the five samples of healthy *P. bungeana* tissues (Figure 4C). Unique OTUs (933, 9.58%) were most abundant in HL samples and least abundant in HR samples (142, 1.06%). A total of 529 (2.60%), 636 (4.04%), and 881 (7.92%) OTUs were present in the HFRS, HRS, and HS samples, respectively. However, nine OTUs (0.48%) were shared across the different compartments of unhealthy *P. bungeana* plants (Figure 4D). Specifically, a total of 545 (35.37%), 178 (9.45%), 545 (28.94%), 203 (10.78%), and 291 (15.45%) unique OTUs were identified in the UFRS, UL, URS, UR, and US samples, respectively. The number of shared OTUs was abundant in the bulk soil (38.52%) compared to that in the rhizospheric soil (37.33%) and roots (27.88%) but least abundant in the stems (9.18%) and leaves (5.87%) of healthy *P. bungeana* plants (Supplementary Figures S4F–J).

### Abundance of the microbial community in soil (bulk and rhizospheric soils) and plant niches (roots, stems, and leaves) of *P. bungeana*

**Figure 5:**
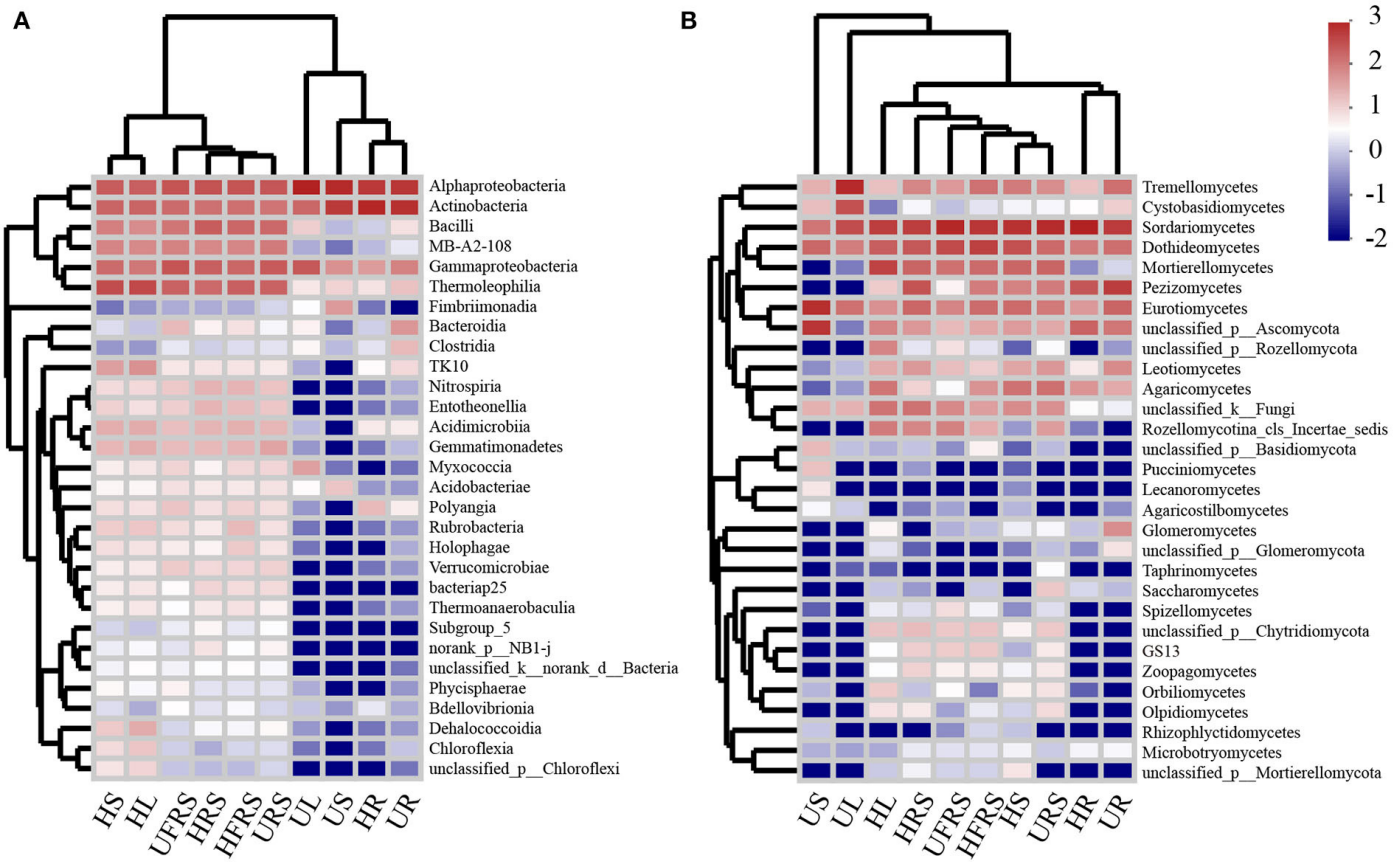
Heatmap analysis of the top 30 classes of **(A)** bacterial and **(B)** fungal communities in the compartments of *P. bungeana* in healthy and unhealthy plants. Abbreviations of variables are given in Figure 1.

The heatmap (Figure 5) and results of sample clustering analysis revealed that the fungal community structures were similar in the bulk and rhizospheric soil samples. However, the composition of the bacterial communities was similar in the HR and UR samples. The dominant classes of bacteria and fungi were different among the HS, US, HL, and UL samples. In addition, the abundance of bacteria and fungi in the rhizospheric soil, bulk soil, and HS and HL samples of *P. bungeana* at different health conditions was significantly higher than that in the other samples (*P* < 0.01).

### Statistical differences in the enrichment of microbial taxa in plants and soils

**Figure 6:**
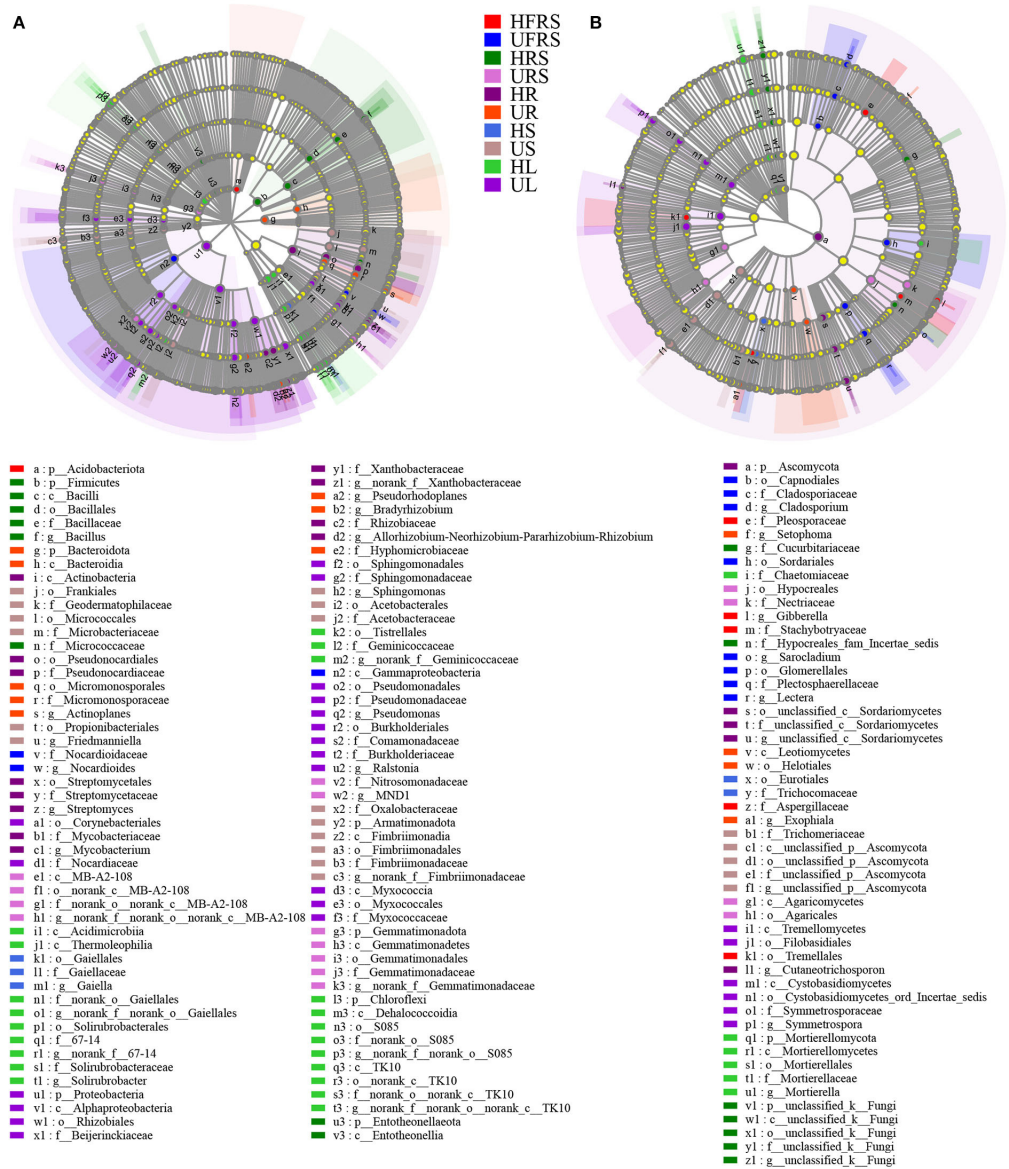
LEfSe (LDA > 4) showing the phylogenetic distribution of **(A)** bacterial and **(B)** fungal microbial taxa (from phylum to genus) in rhizospheric and bulk soils, and different compartments (roots, stems, and leaves) of healthy and unhealthy *P. bungeana* plants. LEfSe analysis was used to search for microbial taxa that were statistically different between groups. The identified microbial taxa are represented as nodes marked with letters in the LEfSe diagram. The circles radiating from the inside to the outside represent the classification levels from phylum to genus. The differently colored regions represent different samples (red, HFRS; dark blue, UFRS; dark green, HRS; pink, URS; dark purple, HR; orange, UR; light blue, HS; brown, US; light green, HL; and light purple, UL). The species with no significant differences (*P* > 0.05) are uniformly colored in yellow, and the different species are colored according to the group. The colors of the nodes correspond to microbial groups that play an important role in the correspondingly colored groups. Abbreviations of variables are given in Figure 1.

LEfSe analyses revealed differences in the composition of the microbial communities in the different plant compartments among healthy and unhealthy plants. Analyses of the bacterial composition revealed that Acidobacteriota was significantly enriched in the HFRS sample (Figure 6A; Supplementary Figure S5A). Nocardioidaceae (from family to genus) and Gammaproteobacteria were significantly enriched in the UFRS sample. Firmicutes (from phylum to genus), Micrococcaceae, and Entotheonellaeota (from phylum to class) were significantly enriched in the HRS sample. The significantly enriched bacteria in the URS sample included MB-A2-108 (from class to genus), Nitrosomonadaceae (from order to family), and Gemmatimonadota (from phylum to genus). Actinobacteria (from class to genus), Proteobacteria (from phylum to genus), and Myxococcia (from class to family) were significantly enriched in the HR sample. Bacteroidota (from phylum to class), Micromonosporales (from order to genus), Pseudorhodoplanes, *Bradyrhizobium* sp., and Hyphomicrobiaceae were enriched in the UR sample. Gaiellales (from order to genus) was enriched in the HS sample. The enriched bacteria in the US samples were Frankiales, Micrococcales, Acetobacterales (all three from order to family), Propionibacteriales, Friedmanniella, Sphingomonas, Oxalobacteraceae, and Armatimonadota (all five from phylum to genus). Acidimicrobiia, Thermoleophilia (from class to genus), Tistrellales (from order to genus), and Chloroflexi (from phylum to genus) were enriched in the HL samples. Proteobacteria (from phylum to genus), Corynebacteriales (from order to family), and Myxococcaceae (from class to family) were enriched in the UR samples. Pleosporaceae, Gibberella, Stachybotryaceae, and Aspergillaceae were significantly enriched in the HFRS samples (Figure 6B; Supplementary Figure S5B), while eight groups of fungi were considerably enriched in the UFRS sample, including Capnodiales (from order to genus), Glomerellales (from order to genus), Sordariales, and *Sarocladium* sp. Cucurbitariaceae, Hypocreales_fam_incertae_sedis, and unclassfied_k_Fungi (from phylum to genus) were significantly enriched in the HRS samples. Hypocreales (from order to family) and Agaricomycetes (from class to order) were significantly enriched in the URS samples, while Ascomycota (from phylum to genus) was significantly enriched in the HR samples. A total of four groups of fungi, namely, Setophoma, Leotiomycetes (from class to order), Helotiales, and Exophiala were significantly enriched in the UR samples. Eurotiales (from order to family) was significantly enriched in the HS samples. Trichomeriaceae and unclassified_p_Ascomycota (from class to genus) were significantly enriched in the US samples. Chaetomiaceae and Mortierellomycota (from phylum to genus) were significantly enriched in the HL samples, while Tremellomycetes (from class to order) and Cystobasidiomycetes (from class to genus) were significantly enriched in the UL samples.

### Relationship between environmental characteristics and microbial communities in rhizospheric and bulk soils of *P. bungeana*

**Figure 7:**
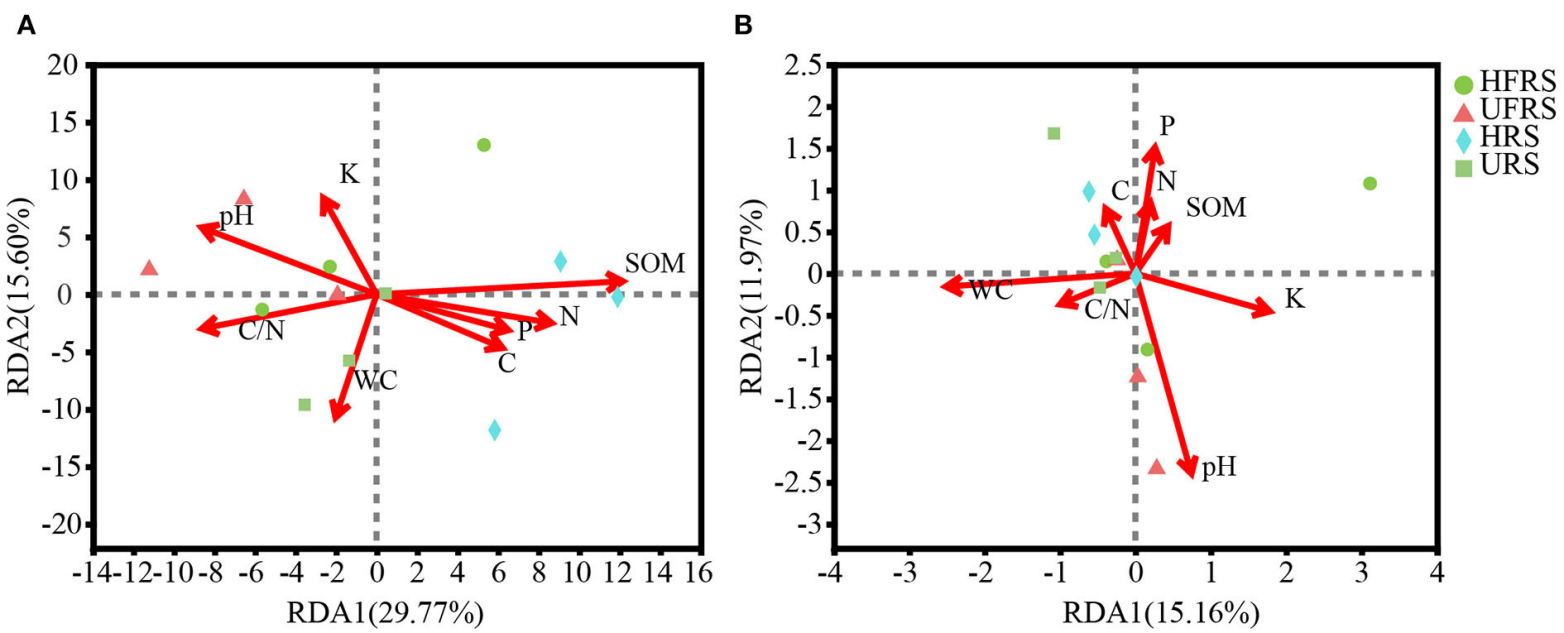
RDA showing the association between edaphic parameters and the composition of **(A)** bacterial and **(B)** fungal communities at the OTU level in the rhizospheric and bulk soils of healthy and unhealthy *P. bungeana* plants. The length of the arrows correspond to the variance that can be explained by the edaphic variable, while the direction indicates an increase in the magnitude of the edaphic variable. The OTUs were clustered using a sequence similarity threshold of 97%. Abbreviations of variables are given in Figure 1.

The pH of the rhizospheric and bulk soils of healthy and unhealthy *P. bungeana* plants ranged from 8.08 to 8.19, indicating that all the tested soils were alkaline. The SOM, TN, TK, and TP contents in the soil of *P*. *bungeana* were at medium levels, according to China Soil Standard protocols for forest regions (LY/T 1232–2015, LY/T 1234–2015, and LY/T 1228–2015, in Chinese). The rhizospheric soil of healthy *P. bungeana* plants had the highest TC content (15.37 g/kg), while the rhizospheric soil of unhealthy *P. bungeana* had the highest C/N (27.33), and the WC levels were approximately 10 g/kg in the rhizospheric and bulk soils of both healthy and unhealthy *P. bungeana* plants (Table 1). RDA demonstrated that the bacterial and fungal communities in the rhizospheric and bulk soils of *P. bungeana* at different health conditions were separated according to the primary environmental characteristics, including SOM, pH, TN, TP, TN, TC, WC, and C/N (Figure 7). The results of the Mantel test revealed that WC and pH were correlated with the microbial communities (*P* < 0.05), and that the bacterial community structure was significantly affected by the SOM content (*P* < 0.01). However, the TN, TP, TK, and TC had no significant effect on microbial community structures in the rhizospheric and bulk soils of healthy and unhealthy *P. bungeana* plants (Table 2).

**Table 1:** Rhizospheric and bulk soil properties of *P. bungeana*.

**Soil property**	**HFRS (M ±SD)**	**UFRS (M ±SD)**	**HRS (M ±SD)**	**URS (M ±SD)**
SOM (g/kg)	16.29 ± 0.52	15.80 ± 0.40	17.42 ± 0.27	16.12 ± 0.57
TN (g/kg)	0.67 ± 0.02	0.52 ± 0.06	0.85 ± 0.19	0.43 ± 0.04
TP (g/kg)	0.66 ± 0.05	0.71 ± 0.17	0.86 ± 0.15	0.62 ± 0.10
TK (g/kg)	15.57 ± 0.04	14.52 ± 0.11	14.19 ± 0.04	14.01 ± 0.14
TC (g/kg)	12.61 ± 0.91	12.45 ± 1.13	15.37 ± 3.11	11.64 ± 0.71
C/N	19.16 ± 1.28	24.25 ± 1.03	18.24 ± 1.05	27.33 ± 0.97
WC (g/kg)	10.44 ± 1.46	10.09 ± 0.44	11.36 ± 0.58	12.1 ± 0.36
pH	8.16 ± 0.08	8.19 ± 0.08	8.08 ± 0.03	8.09 ± 0.06

**Table 2:** Correlation between microbial communities and environmental factors.

**Environmental factors**	**Bacteria**		**Fungi**	
	**R**	***P***	**R**	***P***
TN	0.3758	0.112	0.0778	0.654
SOM	0.7154	0.007^**^	0.0521	0.887
TP	0.2357	0.284	0.2539	0.266
TK	0.3393	0.154	0.3903	0.077
WC	0.5393	0.031^*^	0.7579	0.007^**^
pH	0.4993	0.04^*^	0.6961	0.002^**^
TC	0.2713	0.27	0.0766	0.545
C/N	0.3963	0.129	0.1258	0.639

Correlations between microbial communities and environmental factors were calculated using the Mantel test based on the Bray–Curtis distance algorithm (999 permutations). The degree of correlation is shown as the R and *p*-values. Significance level:

* *P* < 0.05;

** *P* < 0.01.

## Discussion

In this study, the endangered conifer species, *P. bungeana*, in the Millennium Xiulin forest of the Xiong'an New Area was selected to study the community structure and variations in the bacterial and fungal communities in soils (bulk and rhizospheric soils) and niches in different plant compartments (roots, stems, and leaves), with the aim of elucidating the changes in the related microbial communities of trees at different health conditions. The results can provide important insights for preserving the health and promoting the sustainable development of this tree species in plantation forests.

Plant niches are significantly correlated with bacterial communities, whereas fungal communities exhibit a different trend (Devin et al., 2016; Zheng and Gong, 2019). The endospheric microbiome is affected by pathogenic infections and potentially affects microbial composition and function (Bulgari et al., 2011; Douanla-Meli et al., 2013). We observed that the bacterial diversity in the rhizospheric and bulk soils and in the stems and leaves of healthy *P. bungeana* plants was significantly higher than that in the roots of both healthy and unhealthy *P. bungeana* plants and in the stems and leaves of unhealthy *P. bungeana* plants. By contrast, there were no significant differences in the bacterial diversity among the rhizospheric soil (HRS and URS), bulk soil (HFRS and UFRS), HS, and HL samples. Fungal abundance in the HS and HL samples was higher than that in the soil (bulk and rhizospheric soil), root (UR and HR), US, and UL samples. The microbial communities in the US and UL samples differed significantly from those of the other samples. The majority of endophytic microorganisms in the HS and HL samples could have been derived from rainwater, air, insects, or the spread of hyphae or spores, in comparison to the microbial communities in the bulk and rhizospheric soil samples and other plant compartments (UR, HR, US, and UL). In addition, the majority of root endophytic communities are derived from the rhizospheric soil and may have experienced strict biological tissue selection prior to colonization in the roots (Compant et al., 2016; Wei and Ashman, 2018). Furthermore, interactions with the biochemical products of *P. bungeana* may promote the reciprocal relationship between the microorganisms in the stems and leaves of healthy *P. bungeana* plants. By contrast, the presence of pathogenic microorganisms in the stems and leaves of unhealthy *P. bungeana* may greatly prevent supplementation by other microbial communities (Cregger et al., 2018; Liu et al., 2018).

The results of the co-occurrence network (CoNet) analysis revealed that the top 20% of fungal genera characterized in healthy plants showed no significant negative interactions (Supplementary Figure S6B). However, the microbes *Actinophytocola* sp. (Fawzia et al., 2018), *MND1, Microvirga* sp., *Nitrospira* sp., *Paenibacillus* sp., *Sp*. Sp., *Pseudorhodoplanes* sp., *Gaiella* sp., *Blastococcus* sp., *Solirubrobacter* sp., *Nocardioides* sp., *Rhodomicrobium* sp., and *Agromyces* sp. in the roots showed a strong negative correlation (Supplementary Figure S6A). The endemic bacteria in the US, UL, and UR samples of unhealthy plants, including *Methylobacterium-Methylorubrum* sp., *Br*. Sp., *Pedomicrobium* sp., *Modestobacter* sp., *Ps*. Sp., *N*. sp., *Amnibacterium* sp. (Jiang et al., 2018), *G*. sp., *Steroidobacterium* sp., *Mycobacterium* sp., *Actinoplanes* sp., *Friedmanniella* sp., and *Jatrophihabitans* sp. (Supplementary Figure S6C), along with the endemic fungi in the US and UL samples, including *Bradymyces* sp., *Pyrenochaetopsis* sp., *Fusarium* sp., *Symmetrospora* sp., *Aspergillus* sp., *Plectosphaerella* sp., *Gibellulopsis* sp., *Gibberella* sp., *Pecter asp*., *Schizothecium* sp., *Clonostachys* sp., *Strelitziana* sp., *Talaromyces* sp., *Schizothecium* sp., *Volutella* sp., *Filobasidium* sp., *Fusicolla* sp., and *Paracylindrocarpon* sp. (Supplementary Figure S6D), and other dominant bacterial genera in soils, plant tissues, and environmental samples showed a negative correlation, resulting in a strong competitive relationship.

The age of the *P. bungeana* plants selected in this study ranged from 2 to 3 years. Owing to the short period of forest establishment, the plant tissues and the microbial community of the local forest soil are immature, and most of the microorganisms in the tissues may have originated from the caulosphere, phyllosphere, or anthosphere (Zheng and Gong, 2019). In addition, the microbial community of *P. bungeana* may be shaped by its tissue structure, physicochemical properties, specific metabolic pathways, and unique gene products of the host plant, making it possible for the plant niches to recruit certain specific microbes (Horton et al., 2014). Finally, potent abiotic factors, including nutritional availability and exposure to light, can result in marked differences in the microbial communities within the stems and leaves of healthy *P. bungeana* plants (Cregger et al., 2018). The rarefaction curves delineated by the OTUs in soils and tissues were highly uneven, which could be attributed to the uneven microbial colonization of plant roots and inner rings (Bais et al., 2006; Lugtenberg and Kamilova, 2009).

The bacterial and fungal communities in the soil (bulk and rhizospheric soils), and niches in the different compartments (roots, stems, and leaves) of *P. bungeana* differed at the OTU level. A total of 180 bacterial OTUs and 44 fungal OTUs were shared by the samples obtained from the soil and different ecological niches of healthy *P. bungeana* plants. The number of OTUs was higher in the stems and leaves, indicating that most of the microbial communities in stems and leaves could have originated from aerospores. A total of 31 bacterial and nine fungal OTUs were shared among the samples obtained from soils and different ecological niches of unhealthy *P. bungeana* plants, suggesting that the differences in the health conditions of plants altered the diversity and composition of microbial communities. Similar results have also been reported in *Pisum sativum*, and the study demonstrated that the composition and diversity of endophytic microbes were adversely affected in the majority of unhealthy plants (Yu et al., 2012).

A number of fungal OTUs shared by all samples from healthy plants were significantly higher than those of unhealthy plants, indicating that the health conditions of the plants had a pronounced effect on the fungal community. Furthermore, few fungal OTUs were shared among the samples collected from the aboveground sites (stems and leaves), while the fungal OTUs in the samples obtained from underground parts (bulk soil, rhizospheric soil, and roots) were similar. It is, therefore, speculated that the illness of *P. bungeana* could be attributed to a “top-down” disease, such as needle cast, brown blotch, or *Hypoderma desmazieri* Duby, suggesting that quarantine and management strategies should be strengthened, combined with biochemical control measures for eliminating pathogens.

### Niche preference for the microbiome of *P. bungeana*

Plant-specific traits, including internal tissue structure, plant health status, and genetic factors (Lebeis et al., 2015), can affect the abundance and differentiation of microbial communities (Zheng and Gong, 2019). It is widely recognized that soil microbes can successfully colonize host plants, and the host plant successfully attracts microbes from the soil to colonize the root plane through root exudates. Some rhizospheric microbes that are able to successfully colonize the roots pass through the plant endodermis and middle column sheath, reach the xylem vessel, and finally form the endophytic community (Compant et al., 2009). A previous study reported that there is a certain association between endophytes and soil microbes (Cocking, 2003). The results of this study indicated that there are differences and inevitable connections between the microbial composition and diversity in different niches. For instance, Proteobacteria and Actinobacteria were the most abundant bacterial phyla in the soil (bulk and rhizospheric soil) samples and niches in different compartments (roots, stems, and leaves) of healthy and unhealthy *P. bungeana* plants, suggesting that the soils and plant niches are linked in terms of microbial transmission. Microbes also have a certain amount of tolerance and are able to grow and reproduce in different environments, including the phyllosphere, stems, and other compartments. Owing to their high tolerance potential or the need for niche overlap, soil microflora are capable of co-existing in aboveground tissues to a greater extent (Whipps et al., 2008).

Proteobacteria and Actinobacteria are the most abundant bacterial phyla in *P. bungeana* Beckers et al., 2017; Wallace et al., 2018), and it has been reported that Actinobacteria can colonize any plant tissue (Dinesh et al., 2017). Different tissues of the plant can be colonized by different Actinobacteria, which might be determined by the interactions with the microbes of the host plant (Nimnoi et al., 2010). We observed that the abundance of Actinobacteria was higher in the roots than in other tissues. The rhizospheric soil was abundant in Actinomycetes, which is actively recruited by the roots. Actinomycetes such as *Actinomadura glauciflava* might promote root development by producing indole-3-acetic acid (IAA), or the bacteria may produce siderophores for binding environmental Fe^3+^ for improving nutrient absorption, thereby promoting healthy growth (Madhurama et al., 2014). However, we observed that Firmicutes was more abundant in leaves and stems. This finding is in agreement with the results of a study on tree peony (*Paeonia* Sect. *Moutan*) and could be related to air transport and root “block” (Yang et al., 2017). At the genus level, *Bacillus* sp., *Actinop*. sp., and *norank_f_norank_o_Gaiellales* were abundant in the soils and tissues of healthy plants (Supplementary Figure S3C). These genera have been detected in several plants and might be beneficial for plant health and growth (Rodrigo et al., 2011; Wei et al., 2021). The dominant bacterial genera in the UL and US samples were *Sp*. sp. and *Me*. sp., and their abundance might protect unhealthy plants against pathogens or suppress bacterial pathogens by secreting certain substances, including phenylalanine ammonia-lyase, β-1, 3-glucanase, and peroxidase (Tomoyuki et al., 2016; Madhaiyan et al., 2017).

Ascomycota, Basidiomycota, and Mortierellomycota were the dominant fungal phyla in the soil (bulk and rhizospheric soil), and niches in different plant compartments (roots, stems, and leaves) of healthy and unhealthy *P. bungeana* plants (Hardoim et al., 2015). The findings were similar to those reported in *Populus trichocarpa* (Allison et al., 2019) and oak (Toju et al., 2013). Ascomycota and Basidiomycota are widely distributed in different habitats and play an important role in maintaining soil balance and improving plant productivity (Wang et al., 2010; Yang et al., 2019). *Exserohilum* sp., *Le*. sp., *Py*. sp., and *Fusi*. sp., were the dominant genera in healthy plants. These core microbial taxa were shared by soil (bulk and rhizospheric soil) samples and niches in the different compartments (roots, stems, and leaves) of healthy and unhealthy *P. bungeana* plants and were probably vertically transmitted, indicating species conservatism to a certain degree (Wei et al., 2021). The dominant microbial genus in the stems and leaves of unhealthy *P. bungeana* plants was *Fi*. sp., which produces a killer toxin (FC-1) that is highly effective against the opportunistic fungal pathogen *Cryptococcus neoformans* and probably protects the health of the plant (Keszthelyi et al., 2006). However, it is well known that some pathogenic genera, including *Dothistroma septosporum, Do*. *pini*, and *Lecanosticta acicola* belong to the phylum Ascomycota, and can cause plant diseases (Raitelaityte et al., 2017; Van der Nest et al., 2019). The unhealthy *P. bungeana* plants in the study area were highly likely to be infected by these pathogenic fungi, which, in turn, altered the composition of the endophytic microbial community. Microbial interactions play an important role in plant health, and endophytic plant microbes can contribute to the growth and health of organisms through their structural and functional diversity (Santhanam et al., 2015; Agler et al., 2016). Following infections with pathogenic bacteria, specific microorganisms can be enriched in the plant tissue for the continual inhibition of pathogenic bacterial disease *via* the secretion of certain compounds or the expression of synthetic gene clusters (Carrión et al., 2019). As aforementioned, the microbial communities in the tissues of *P. bungeana* showed differences at different health conditions (e.g., *Sp*. sp., *Me*. sp., and *Fi*. sp.), which together protected the plant from internal and external stressors *via* several unknown functional traits.

The microbial diversity and abundance were lowest in the roots, which could be related to the source of endophytic microbes and their growth and expansion. A large number of microorganisms in the soil and rhizosphere were challenged during entry into roots owing to the barrier effect. By contrast, there were abundant microorganisms in the stems and leaves due to the invasion of aerial spores (Timon et al., 2013). These phenomena highlighted the differences in microbial diversity among plant-related habitats (Pinto et al., 2014; Peay et al., 2016; Hassani et al., 2018), and the lower microbial diversity in stems and leaves of unhealthy *P. bungeana* plants could allow the invasion of pathogenic fungi, including *St*. sp. and *Pestalotiopsis* sp., resulting in the development of resistance to other groups of microorganisms.

### Effects of soil abiotic factors on soil microbial communities

It is generally believed that plants have specific core microflora that dynamically responds to the environment, including soil properties (Podolich et al., 2015). Soil microbiome is a key player in the maintenance of the health of soil ecosystems, and soil properties are the main factors that affect the existence and diversity of soil microorganisms (Thiem et al., 2018; Whitaker et al., 2018). It has been proposed that soil type is one of the main driving factors for rhizospheric microbial communities (Hinsinger et al., 2009; Yaish et al., 2016). Soil microorganisms can promote material flow and energy conversion by decomposing nutrient substances to gradually improve soil quality (Bouchez et al., 2016). In this study, soil nutrients were at medium or medium to low levels. The distribution of the soil bacterial and fungal communities of *P. bungeana* was significantly affected by the WC (water content) and pH (*P* < 0.05). The soil WC was closely related to the expression of certain functional genes of the microbes (Bahram et al., 2018) and was the main factor affecting the structure and function of soil microbial communities. Water can affect soil aeration conditions, and good soil aeration conditions are the basis for the survival of most aeromicrobes (Peralta et al., 2012). Soil pH has a substantial effect on the composition of soil microbial communities, and the acid–base property of soils has a highly significant effect on the soil microbial composition (Lauber et al., 2009; Rousk et al., 2010). In addition, the SOM significantly affected the soil bacteria of *P. bungeana* (*P* < 0.01). SOM is an important soil fertility index, providing crops with required substances and improving the physical and chemical conditions of soils. It is one of the most important soil nutrients that can directly affect the soil structure, air permeability, and other properties. The SOM content was significantly positively correlated with the improvement in microbial activity and developmental potential (Bastida et al., 2015). However, no significant correlation was found between soil TN, TP, TK, TC, and C/N and the distribution of microbial communities in this study, which could be attributed to the low content of these elements in our samples.

## Conclusion

This study highlighted the fundamental effects of the health status of *P. bungeana* on the microbial communities in different niches of *P. bungeana* in the plantation forests in the Xiong'an New Area in China. The most significant finding of this study is that the health of the plants had significant effects on the endophytic bacterial and fungal communities in the stems and leaves of *P. bungeana*. The results of this study demonstrated that there were no differences in the bacterial and fungal alpha diversities of the samples of bulk soil, rhizospheric soil, and roots between healthy and unhealthy *P. bungeana* plants. The microbial diversity was found to be higher in the stems and leaves of healthy *P. bungeana* plants. In addition, this study confirmed that the richness of the microbial communities in the aboveground parts (stems and leaves) of healthy *P. bungeana* plants was much higher than that of the unhealthy plants. By contrast, there were almost no differences in the richness of the microbial communities in the underground parts (bulk soil, rhizospheric soil, and roots), suggesting that the possible cause of illness was transmitted in a “top- down” manner in unhealthy plants. Altogether, the results provided a scientific basis for further studies on the mechanism of the “aboveground–underground” microbial interactions, which can aid in promoting the healthy and sustainable development of the Millennium Xiulin forest in the Xiong'an New Area.

## Data availability statement

The datasets presented in this study can be found in online repositories. The names of the repository/repositories and accession number(s) can be found below: https://www.ncbi.nlm.nih.gov/, PRJNA821431; https://www.ncbi.nlm.nih.gov/, PRJNA821418.

## Author contributions

JY: writing—original draft. JY, HL, YG, and AM: methodology, software, data curation, and validation. CW and MW: soil sampling and experiment. ZY and JL: conceptualization, supervision, funding acquisition, and review and editing. All authors contributed to the article and approved the submitted version.

## Funding

This study was supported by the Hebei Science and Technology Innovation Base Project (216Z2903G), the National Key Research and Development Project of China (2018YFC0506900), the National Natural Science Foundation of China (31672365), the National Natural Science Foundation of China (42071257), and the Postgraduate Innovation Foundation of Hebei (CXZZBS2022071).

## Conflict of interest

The authors declare that the research was conducted in the absence of any commercial or financial relationships that could be construed as a potential conflict of interest.

## Publisher's note

All claims expressed in this article are solely those of the authors and do not necessarily represent those of their affiliated organizations, or those of the publisher, the editors and the reviewers. Any product that may be evaluated in this article, or claim that may be made by its manufacturer, is not guaranteed or endorsed by the publisher.
