# Supplementary material for: Microbial community structure and niche differentiation under different health statuses of Pinus bungeana in the Xiong'an New Area in China
Source: Front Microbiol. 2022 Sep 2;13:913349. doi: 10.3389/fmicb.2022.913349 (PMC9481298; doi:10.3389/fmicb.2022.913349)
Supplement: Supplementary file 1 [file Data_Sheet_1.docx]

**Microbial community structure and niche differentiation under different health statuses of *Pinus bungeana* in the Xiong'an New Area in China**

Jia Yang^1^, Abolfazl Masoudi^1^, Hao Li^1^, Yifan Gu^2^, Can Wang^1^, Min Wang^1^, Zhijun Yu^1^*, Jingze Liu^1^*

^1^Hebei Key Laboratory of Animal Physiology, Biochemistry and Molecular Biology, Hebei Collaborative Innovation Center for Eco-Environment, Ministry of Education Key Laboratory of Molecular and Cellular Biology, College of Life Sciences, Hebei Normal University, Shijiazhuang, Hebei 050024, China

^2^School of Geographic Sciences, Hebei Normal University, Shijiazhuang, Hebei 050024, China

* Correspondence:

Corresponding authors: Zhijun Yu, Jingze Liu

E-mail addresses: yuzhijun@hebtu.edu.cn (Z. Yu); liujingze@hebtu.edu.cn (J. Liu).


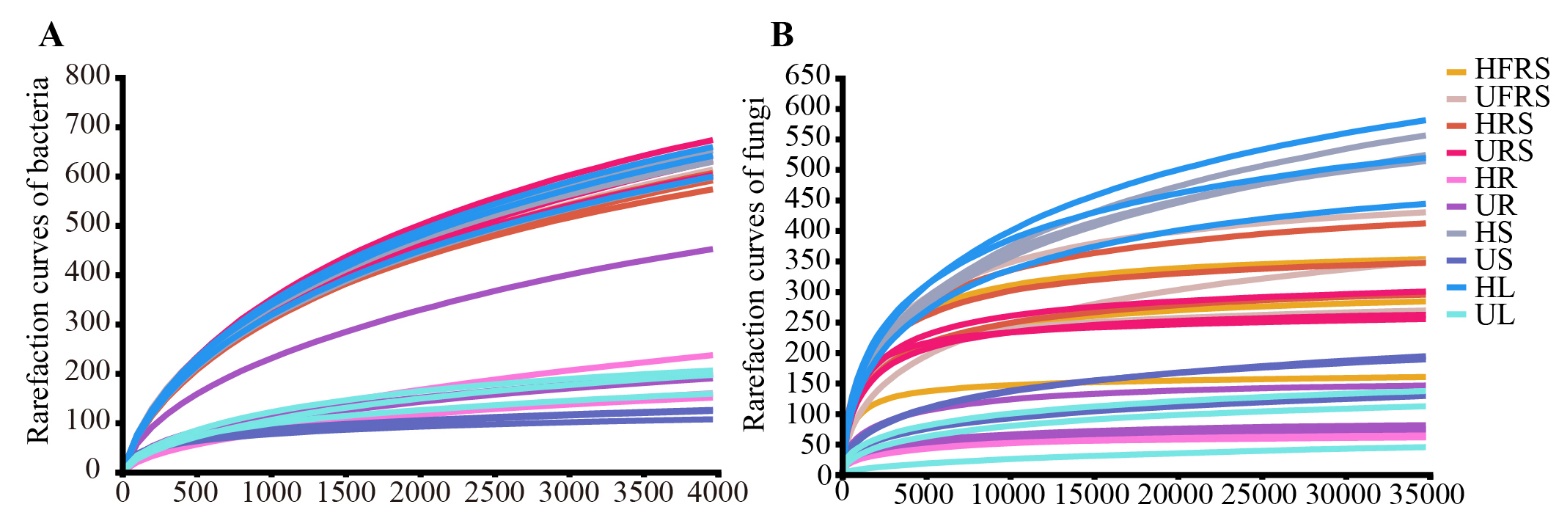


**Figure S1** Alpha diversity of the bacterial and fungal communities in healthy and unhealthy *P. bungeana* plants. The pictures show (**A**) bacterial OTU richness and (**B**) fungal OTU richness rarefaction curves. Abbreviations: healthy bulk soil (HFRS), healthy rhizosphere soil (HRS), healthy roots (HR), healthy stems (HS), healthy leaves (HL), unhealthy bulk soil (UFRS), unhealthy rhizosphere soil (URS), unhealthy roots (UR), unhealthy stems (US), and unhealthy leaves (UL).


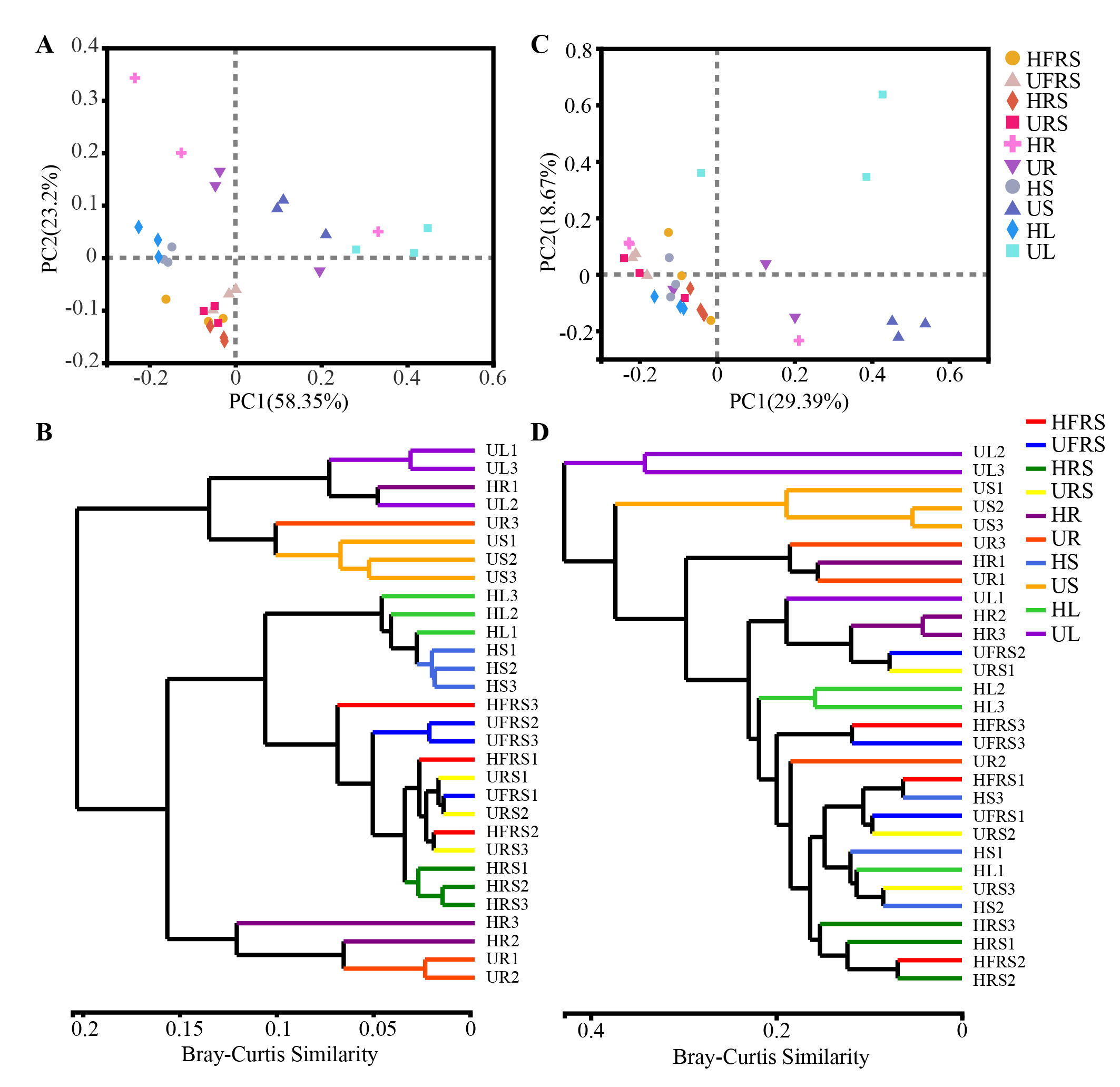


**Figure S2** Composition of the microbial communities in rhizospheric and bulk soils, and different compartments (roots, stems, and leaves) of healthy and unhealthy *P. bungeana* plants was analyzed by PCoA and dendrograms constructed using the hierarchical clustering algorithm at phylum/class levels. (**A**) PCoA of the bacterial communities. (**B**) Hierarchical clustering of the bacterial dendrograms. (**C**) PCoA of the fungal communities. (**D**) Hierarchical clustering of the fungal dendrograms. PCoA was based on the rarefaction curve of the minimum sample sequence number. Hierarchical clustering was based on the Bray–Curtis distance and superimposed on PCoA plots. The OTUs were constructed using a sequence similarity threshold of 97%. Abbreviations of variables are given in Figure S1.

At the 197 order levels (Figure. S3A), the dominant order of bulk soil and rhizosphere soil was Rhizobiales (10.61%), Burkholderiales (10.15%), Bacillales (10.76%). Pseudonocardiales (HR=30.10%, UR=24.36%) had the highest content in root samples, Sphingomonadales (US=18.73%, UL=20.78%) had more unhealthy stems and leaves than other samples, and Gaiellales (HS=13.06%, HL=12.57%) were the dominant order of HS and HL. Among the 314 families (Figure. S3B), Bacillaceae (9.56%) was the dominant family in bulk soil and rhizosphere soil, the dominant family in root samples was Pseudonocardiaceae (HR=30.97%, UR=24.36%), and the dominant family in HS and HL was norank_o_Gaiellales (HS =6.64%, HL=7.12%). Beijerinckiaceae (US=14.28% UR=36.26%), Sphingomonadaceae (US=18.73% UR=20.78%) had the highest content in unhealthy stems and leaves. At the 565 genus levels (Figure. S3C), *Bacillus* (9.46%) had the highest content in rhizosphere soil and bulk soil, *Actinophytocola* (HR=29.23%, UR=22.55%) had the highest content in roots, norank_f_norank_o_*Gaiellales* (HS=6.64%, HL= 7.12%) was the dominant genus of healthy stems and leaves, the dominant genus of US was *Sphingomonas* (17.52%), and the dominant genus of UL was *Methylobacteriun-Methylorubrum* (33.00%).

At the 107 orders (Figure. S3D), the dominant orders of HFRS were Hypocreales (22.41%), Pleosporates (22.19%), and Mortierellales (8.27%); the dominant orders of UFRS were Hypocreales (20.92%), Glomerellales (17.29%), Sordariales (13.65%); 19.19%) Pezizales (15.94%) was the dominant order of HRS; the main order of URS was Hypocreales (33.42%); unclassified_c_Sordariomycetes (28.17%), Pezizales (29.88%) were the dominant orders of HR and UR respectively; Hypocreales (26.00%), Pleosporates (11.43%) was the predominant order of HS, US predominant order was unclassified_p_Ascomycota (36.11%), Chaetothyriales (40.43%), Mortierellales (25.08%) was the predominant order of HL, and the main order of UL was Filobasidiales (49.51%). At the 242 families (Figure. S3E), Pleosporaceae (15.38%), Plectosphaerellaceae (15.91%), Mortierellaceae (9.79%) and Nectriaceae (22.67%) were the dominant families of HFRS, UFRS, HRS and URS respectively, and the dominant family of HR and UR was unclassified_c_Sordariomycetes (28.17%), Tuberaceae (22.08%); Nectriaceae (10.85%), Trichomeriaceae (38.62%) were the dominant families of HS and US respectively. Mortierellaceae (25.08%), Filobasidiaceae (49.50%) were the dominant families of HL and UL respectively. At the 485 genus (Figure. S3F), *Exserohilum* (13.5%) and *Lectera* (10.51%) accounted for the most in HFRS and UFRS, respectively; *Pyrenochaetopsis* (11.78%) and *Fusicolla* (11.57%) were the dominant genus for HRS and URS, respectively; the dominant genus for HR was unclassified_c_*Sordariomycetes* (28.17%); Tuber (22.06%) was the dominant genus of UR; the dominant genus of HS and HL were *Mortierella* (HS=9.46%, HL=25.08%). *Bradymyces* (37.92%), *Filobasidium* (49.50%) were US, UL dominant genus.


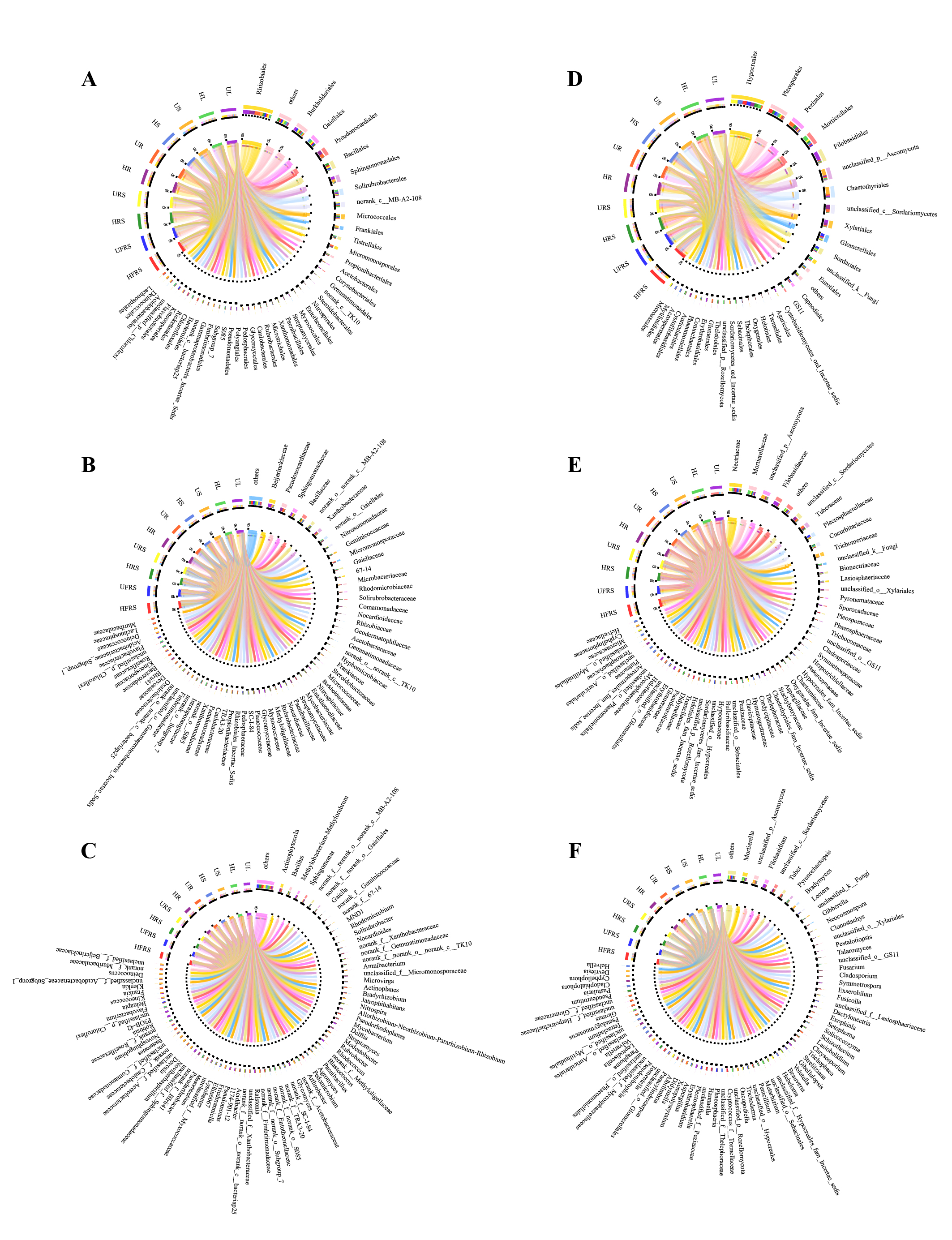


**Figure S3** Relative abundance of all the bacteria and fungi at the order, family, and genus levels in rhizospheric and bulk soils and different compartments (roots, stems, and leaves) of healthy and unhealthy *P. bungeana* plants. The data are visualized using Circos plots, in which the thickness of the ribbons represents the abundance of the taxa. The absolute tick above the inner segment and the relative tick above the outer segment represent the read abundances and relative abundance of the taxa, respectively. The Circos diagram depicts the proportion of core microbiota in each compartment and reflects the proportion of each dominant species in the different samples. Bacterial communities in the Circos plot at the (**A**) order, (**B**) family, and (**C**) genus levels. Fungal communities in the Circos plot at the (**D**) order (**E**) family, and (**F**) genus levels. The three biological replicates in each group. Abbreviations of variables are given in Figure S1.


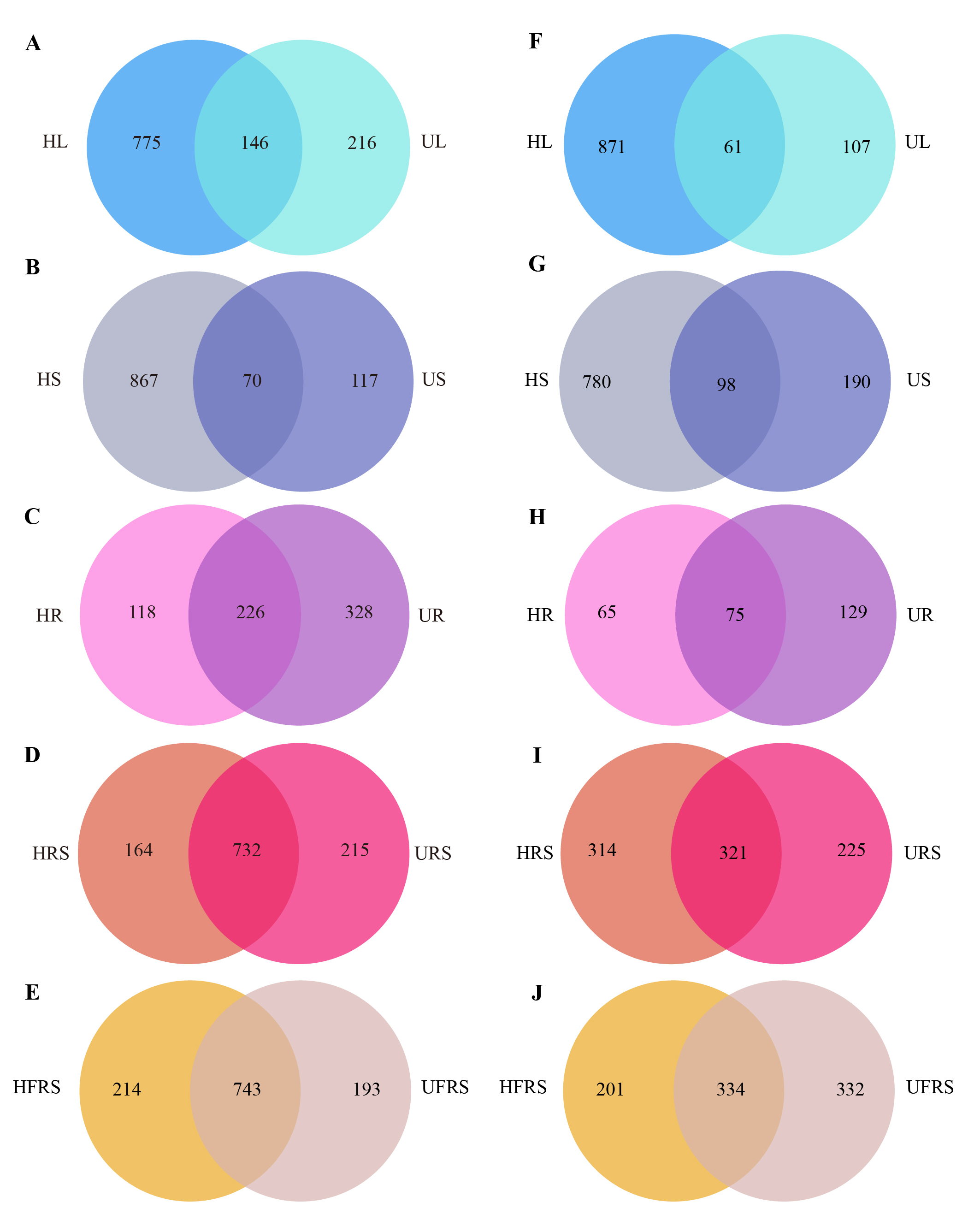


**Figure S4** (**A–E**) Number of bacterial OTUs in the soil and different compartments of healthy and unhealthy *P. bungeana* plants. (**F–J**) Number of fungal OTUs in the different compartment groups. Abbreviations of variables are given in Figure S1.


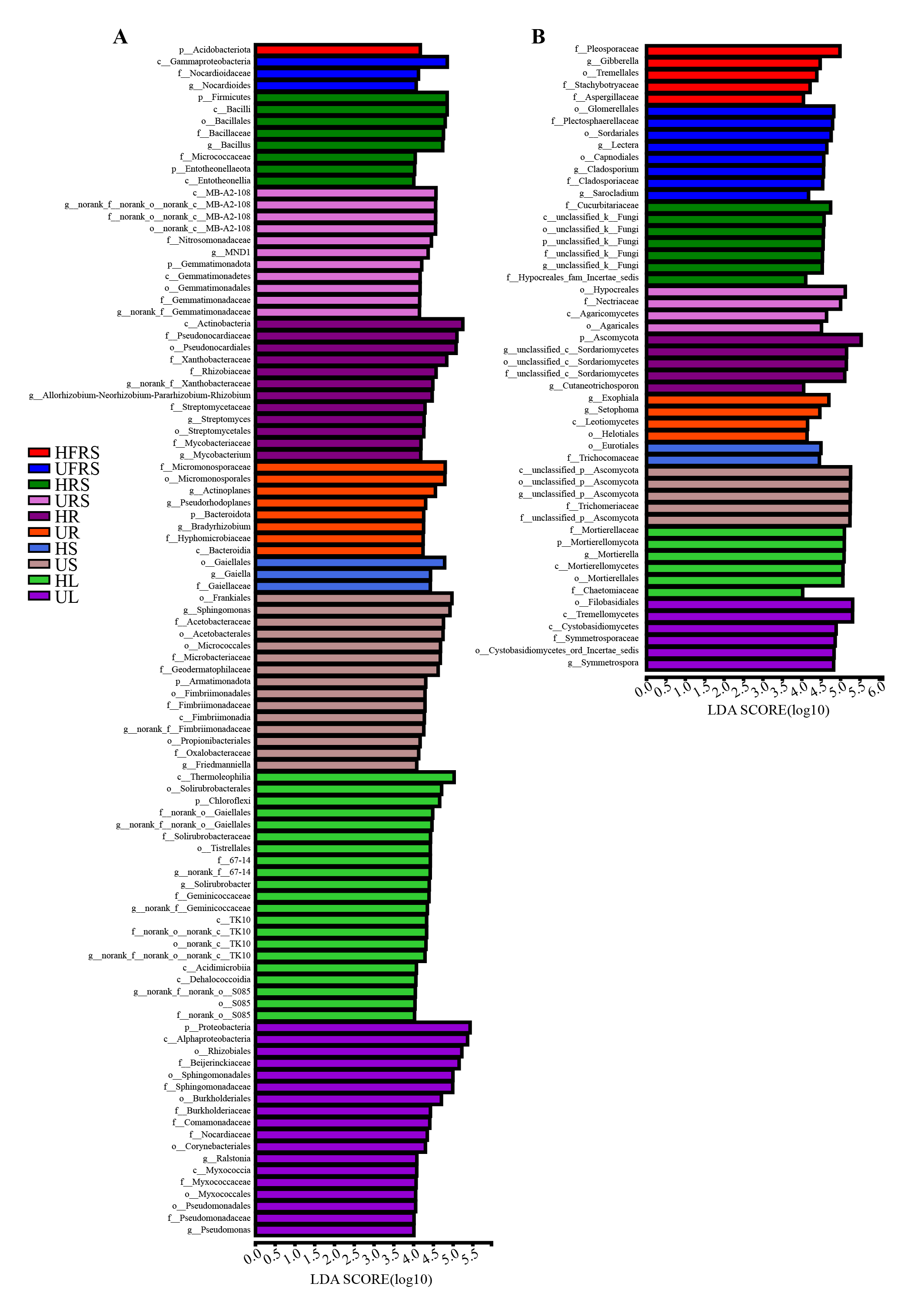


**Figure S5** (**A**) Indicator bacteria with LDA scores ≥ 4 in the bacterial communities associated with rhizospheric and bulk soils, and different compartments (roots, stems, and leaves) of healthy and unhealthy *P. bungeana* plants. (**B**) Indicator fungi with LDA scores ≥ 4 in the fungal communities associated with rhizospheric and bulk soils, and different compartments (roots, stems, and leaves) of healthy and unhealthy *P. bungeana* plants. Abbreviations of variables are given in Figure S1.


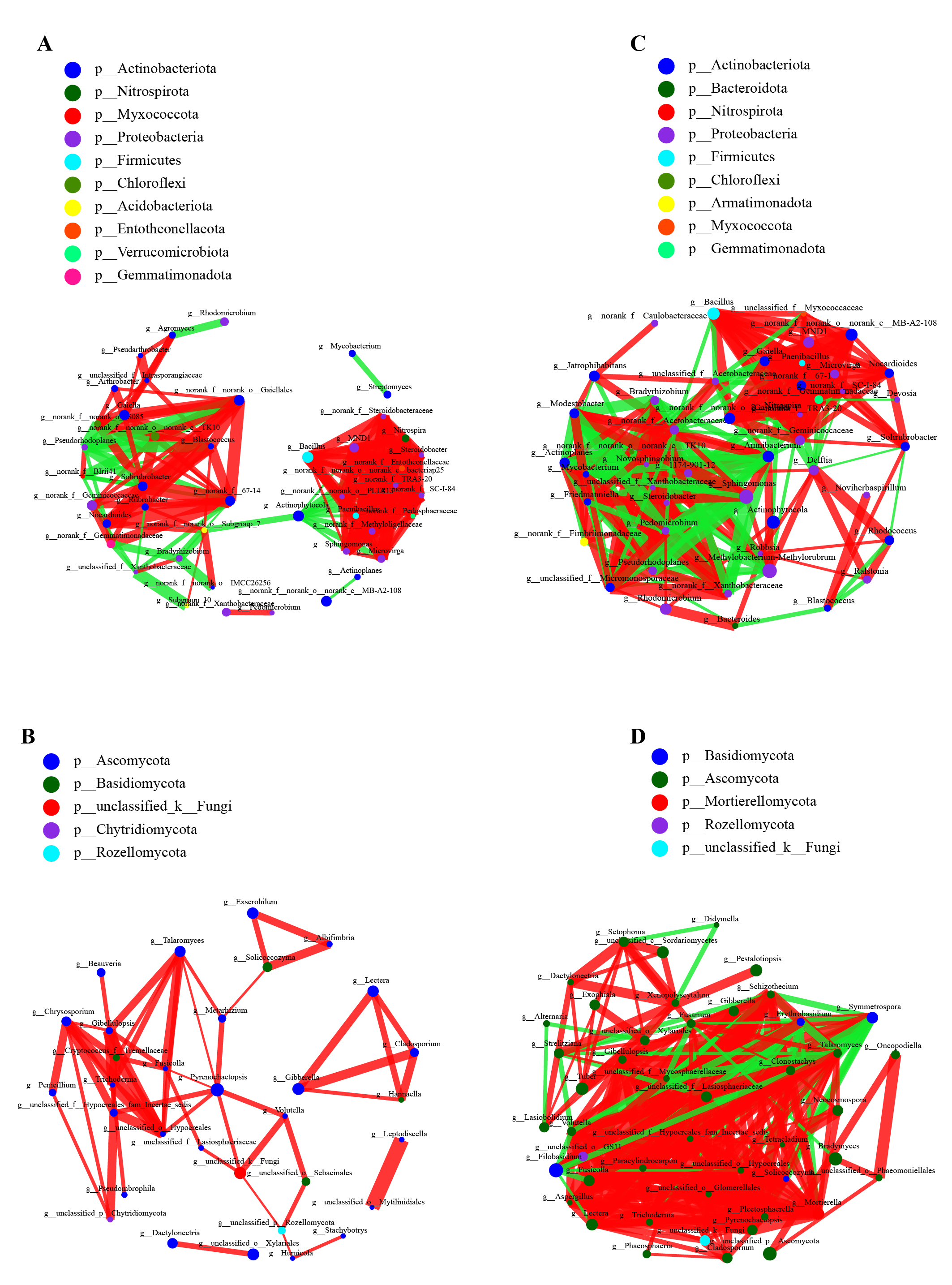


**Figure S6** Microbial network analysis of healthy and unhealthy *P. bungeana* plants. (**A**) Top 20% co-occurring bacteria at the genus level in healthy *P. bungeana* plants*.* (**B**) Top 20% co-occurring fungi at the genus level in healthy *P. bungeana* plants*.* (**C**) Top 20% co-occurring bacteria at the genus level in unhealthy *P. bungeana* plants*.* (**D**) Top 20% co-occurring fungi at the genus level in unhealthy *P. bungeana* plants*.* The sizes of the nodes are proportional to the abundance of the microbial genera. The color of the nodes corresponds to the phylum taxonomic classification. The color of the edges represents positive (green) and negative (red) correlations, while the thickness of the edge is equivalent to the correlation values. Abbreviations of variables are given in Figure S1.
